# Oxetane Grafts Installed Site‐Selectively on Native Disulfides to Enhance Protein Stability and Activity In Vivo

**DOI:** 10.1002/anie.201708847

**Published:** 2017-10-20

**Authors:** Nuria Martínez‐Sáez, Shuang Sun, Davide Oldrini, Pietro Sormanni, Omar Boutureira, Filippo Carboni, Ismael Compañón, Michael J. Deery, Michele Vendruscolo, Francisco Corzana, Roberto Adamo, Gonçalo J. L. Bernardes

**Affiliations:** ^1^ Department of Chemistry University of Cambridge Lensfield Road CB2 1EW Cambridge UK; ^2^ Instituto de Medicina Molecular, Faculdade de Medicina Universidade de Lisboa Avenida Professor Egas Moniz 1649-028 Lisboa Portugal; ^3^ GSK Vaccines Via Fiorentina 1 53100 Siena Italy; ^4^ Departamento de Química, Centro de Investigación en Síntesis Química Universidad de La Rioja 26006 Logroño Spain; ^5^ Cambridge Centre for Proteomics, Cambridge Systems Biology Centre Department of Biochemistry University of Cambridge Tennis Court Road Cambridge CB2 1QR UK

**Keywords:** antibodies, disulfides, immunogenic proteins, oxetanes, stapling

## Abstract

A four‐membered oxygen ring (oxetane) can be readily grafted into native peptides and proteins through site‐selective bis‐alkylation of cysteine residues present as disulfides under mild and biocompatible conditions. The selective installation of the oxetane graft enhances stability and activity, as demonstrated for a range of biologically relevant cyclic peptides, including somatostatin, proteins, and antibodies, such as a Fab arm of the antibody Herceptin and a designed antibody DesAb‐Aβ against the human Amyloid‐β peptide. Oxetane grafting of the genetically detoxified diphtheria toxin CRM_197_ improves significantly the immunogenicity of this protein in mice, which illustrates the general utility of this strategy to modulate the stability and biological activity of therapeutic proteins containing disulfides in their structures.

The rational modification of the structure of peptides and proteins offers a wide range of opportunities for the modulation of their biological activity.^1^ Many efforts have been made to develop strategies that induce such conformational changes and modulation. Towards this end, macrocyclization and stapling have emerged as useful tactics to chemically manipulate peptides and proteins, increasing their proteolytic stability, cell permeability, and producing changes in polarity, binding activity, and pharmacokinetic properties.^2^ During the last years, different approaches have been developed for the covalent tethering of the side chains of natural or non‐canonical amino acids.^2^ Considering natural residues, cysteine (Cys) has been the residue of choice for stapling through alkylation,^3^ arylation,^4^ cycloaddition,4b and disulfide forming reactions both at native^5^ or engineered^6^ Cys residues. More recently, nitrogen arylation has also been shown to be a useful strategy for macrocyclization of lysine residues on peptides.^7^ Otherwise, efficient macrocyclization of linear peptides through the formation of an oxadiazole has also been reported.^8^ However, a large number of stapling/macrocyclization/re‐bridging strategies consist of the introduction of non‐canonical amino acids and their subsequent ligation by ring‐closing metathesis,^9^ lactamization,^10^ or cycloaddition reactions.2c, 4b Common to many of these strategies is either the requirement for complicated orthogonal protection procedures, sequence engineering, the appendage of bulky/constrained linkers between the two residues, or the use of organic solvents. These conditions have limited, for instance, the application of such methods for the stapling of residues on intact, full‐length proteins to impart structural conformational constraints leading to enhanced stability and activity. Thus, there remains a need for simple and robust strategies for stapling native peptides and proteins.

Herein we report a method for site‐selective peptide and protein stapling through the bis‐alkylation of the sulfhydryl side chain of Cys residues resulting from disulfide reduction, using commercially available 3,3‐bis(bromomethyl)oxetane **1** (Figure 1). Oxetanes have become common motifs in drug design due to their ability to modulate parameters including solubility, basicity, lipophilicity, and metabolic stability.^11^ While there are examples of the modification of small peptides with oxetanes,^12^ their incorporation and modulation of the structure and activity of complex biomolecules^13^ remain mostly unexplored.

**Figure 1:**
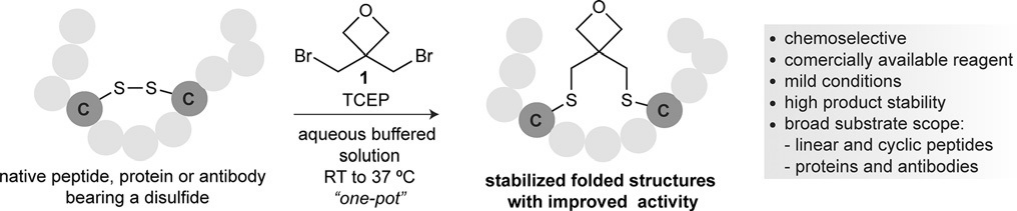
Stabilization of folded structures of peptides and proteins through bis‐alkylation of Cys residues present in the form of a native disulfide using an oxetane graft.

A requirement for a direct method to graft Cys residues present as disulfides on proteins is the compatibility of the reagent with a reducing agent, such as tris(2‐carboxyethyl)phosphine (TCEP). Importantly, we found that a model pentapeptide bearing two Cys residues reacted with **1** in the presence of TCEP to afford the corresponding stapled cyclic peptide in 75 % yield (see the Supporting Information, Figures S2–S5, S34–S39, 48–49 for characterization and discussion of structural features). We then explored the use of **1** to graft two cyclic and biologically relevant peptides: somatostatin **2** and its analogue octreotide **4**, which can be used for imaging and treating neuroendocrine tumors.^14^ Peptides **2** and **4** were reacted simultaneously with TCEP and oxetane **1** in a 1:9 mixture of DMF/H_2_O at 25 °C for 12 h. After HPLC purification, the grafted cyclic peptides **3** and **5** were obtained in 76 % and 81 % yield, respectively (Figure 2 a). The affinity of these derivatives to the natural somatostatin receptor 2 (SSTR2) was experimentally determined by tryptophan fluorescence spectroscopy (Supporting Information, Figure S1). While octreotide **4** and surrogate peptide **5** showed a similar affinity against this receptor, grafted somatostatin **3** displayed improved binding properties, showing a 4‐fold enhancement in *K*_D_ value (Figure 2 b). The improvement of binding activity is a considerable advantage of the incorporation of the oxetane graft when compared, for instance, with the recently reported methylene thioacetal that led to a decrease in binding affinity to SSTR2.3b Interestingly, 0.5 μs MD simulations performed on these derivatives in explicit water and using ff14SB amber force‐field^15^ suggested that octreotide **4** and its stapled derivative **5** presented a similar conformational behavior in solution (Figure 2 d; Supporting Information, Figure S8),^16^ displaying an equilibrium between antiparallel β‐sheet structures and conformations in which the C‐terminal residues form a 3_10_ helix‐like fold, as reported in DMSO solution. In contrast, stapled somatostatin **3** was more rigid and displayed a more defined conformation in solution than **2** (Figure 2 c; Supporting Information, Figures S6 and S7).^17^ In fact, **3** showed a closely related β‐sheet arrangement in solution stabilized by a typical hydrogen bond network, which is apparently ideal for a more efficient binding to the receptor. Finally, analysis of the stability of **3** and **5** both in human plasma as well in the presence glutathione (GSH) showed that the oxetane grafted peptides remain intact under these conditions (Supporting Information, Figures S13–S18).

**Figure 2:**
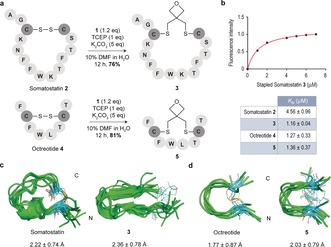
a) Stapling of disulfide‐containing cyclic peptides **2** and **4**. b) Binding affinity studies. *K*_D_ values were determined by tryptophan fluorescence spectroscopy. c),d) Structural ensembles obtained by 0.5 μs MD simulations. The peptide backbone is in green. Carbon atoms of Cys residues as well as of the oxetane moiety are in cyan. The numbers indicate the root‐mean‐square deviation (RMSD) for heavy‐atom superimposition of the backbone with respect to the average structure.

To initially test the potential of using this one‐pot, site‐selective bis‐alkylation oxetane stapling method directly on proteins, we chose thioredoxin (Trx) as a model protein that features a naturally occurring, solvent‐exposed disulfide bond. We could reduce and staple the disulfide bond in a straightforward manner through selective bis‐alkylation with **1** in the presence of TCEP and 10 % DMF in sodium phosphate buffer at pH 8.5. Complete conversion was achieved after 24 h at 37 °C, as confirmed by HPLC‐MS analysis (Supporting Information, Figures S19 and S20). Furthermore, analysis of the CD spectra of the native and stapled Trx‐**1** (Supporting Information, Figure S22) indicated that both molecules present very similar conformational preferences in solution. Although this is supported by MD simulations performed on both proteins in explicit water, the calculations indicate a small increase in flexibility for the peptide backbone of stapled Trx‐**1** (Supporting Information, Figure S11). This result may be explain attending to the greater S−S distance in Thrx‐**1** when compared to the native Trx (4.18 and 2.04 Å, respectively). Finally, we confirmed the suppression of Trx redox activity^18^ through the selective and covalent disulfide stapling (Supporting Information, Figure S21).

Next, we demonstrated the utility of the oxetane graft to build stapled antibodies. First, the exposed disulfide bond tethering the heavy and light chains of a Fab fragment of Herceptin (Fab‐Her), an antibody currently used to treat Her2+ breast cancer patients,^19^ was readily stapled using **1** under aqueous buffered conditions in the presence of TCEP at pH 8.5 and at 37 °C (Figures 3 a,b; Supporting Information, Figures S23 and S24). The oxetane stapled Fab‐Her‐**1**, unlike the disulfide native antibody, was stable under reducing conditions and in human plasma (Supporting Information, Figures S25 and S26). This stability is a key aspect of antibody therapeutics design as thiol‐exchange reactions in plasma lowers efficacy and adds side‐toxicity.^20^ Importantly, a relatively small but significant increase in binding affinity to the Her2 receptor, as determined by bio‐layer interferometry (BLI) experiments (Figure 3 d; Supporting Information, Figure S27), was observed for Fab‐Her‐**1** when compared with the native antibody. Next, we extended our stapling strategy to the antibody DesAb‐Aβ_3‐9_, which was designed to target the region 3–9 of human Amyloid‐β (Aβ42) peptide, the aggregation of which is a hallmark of Alzheimer's disease.^21^ This antibody features a challenging, hindered intra‐domain disulfide typical of VH domains. Of note, complete conversion into the oxetane grafted antibody DesAb‐Aβ_3‐9_‐**1** was achieved using our method (Figure 3 c; Supporting Information, Figures S28 and S29). Owing to the fact that the disulfide is deeply buried, an excess of TCEP (40 equiv) and longer reaction times were required (Supporting Information). Unlike the reduced antibody that readily reacts with thiol‐specific Elman's reagent, the stapled DesAb‐Aβ_3‐9_‐**1** did not react suggesting complete consumption of the reduced Cys during stapling (Supporting Information). Finally, analysis of secondary structural content by CD showed no significant differences between the original and stapled antibodies (Figure 3 e). MD simulations performed on a 3D model of DesAb‐Aβ_3‐9_, previously generated using ABodyBuilder,^22^ and on the stapled derivative DesAb‐Aβ_3‐9_‐**1**, suggest that, although the 3D structure is maintained upon the chemical modification, the oxetane motif provokes a slightly increase in the degree of flexibility (Supporting Information, Figure S31). Collectively, these data demonstrate the suitability of the oxetane motif to staple solvent accessible disulfide bonds on proteins with minimal secondary structure alterations.

**Figure 3:**
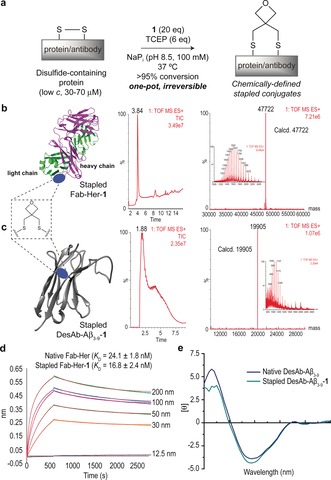
a) Representation of disulfide stapling of native antibody sequences using an oxetane graft. b),c) Total ion chromatogram, combined ion series and deconvoluted mass spectrum reconstructed from the ion series using the MaxEnt algorithm. b) Fab‐Her‐**1** (pdb ID Fab‐Her: 1N8Z) and c) DesAb‐Aβ_3‐9_‐**1** (3D model of DesAb‐Aβ_3‐9_ generated using ABodyBuilder, see main text). d) BLI and fit curves obtained for Fab‐Her‐**1**, together with the derived *K*_D_ constants for Fab‐Her and Fab‐Her‐**1**. e) CD spectra of DesAb‐Aβ_3‐9_ and DesAb‐Aβ_3‐9_‐**1**.

To demonstrate the practical application of our method to therapeutic proteins, we investigated the effects of the selective introduction of the oxetane staple into the genetically detoxified diphtheria toxin CRM_197_, which features four Cys residues in the form of two disulfides. Recently, it has been shown that antibodies against CRM_197_ neutralized diphtheria toxin in HIV infected young individuals.^23^ Furthermore, CRM_197_ is a clinically used carrier in many glycoconjugate vaccines.^24^ Previous structural studies showed that only the disulfide C186‐C201 connecting the fragment A (C domain) and B (T/R domain) of CRM_197_ is selectively reduced in the presence of the highly hindered C461–C471 disulfide upon treatment with dithiothreitol.24b Addition of a slight excess of TCEP to CRM_197_ under aqueous buffered conditions at pH 8.5 and 37 °C, followed by an excess of **1**, led to the introduction of one oxetane graft (Figure 4 a,b and the Supporting Information, Figure S32 for mass spectrometry analysis), presumably at C186–C201 according to our previous findings.^25^ The impact of the installation of the oxetane moiety into CRM_197_ on its structure and thermal stability was studied by CD and differential scanning calorimetry (DSC) analysis, respectively, and compared with the native protein (Figure 4 c–e). We found that both the far and near UV CD spectra of CRM_197_‐**1** were nearly identical to those of CRM_197_, which indicates that the 3D structure is preserved upon the chemical stapling. The DSC curves also corroborate this finding. Although CRM_197_‐**1** exhibited a broader DSC peak when compared to the sharp change in heat capacity, in the range of 40–55 °C for CRM_197_, both proteins present an identical transition midpoint (*T*_m_) of 46 °C.

**Figure 4:**
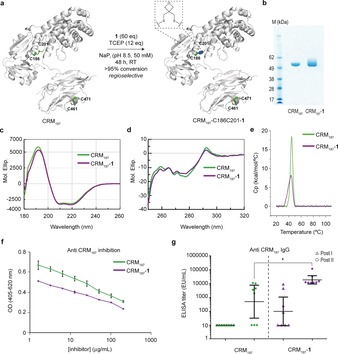
Enhancing the immunogenicity of a protein carrier through disulfide oxetane stapling. a) The functional stapling of CRM_197_ (pdb ID CRM_197_: 4AE0) with **1**. b) SDS‐page of native and stapled CRM_197_ **‐1**. c) Far UV CD spectrum. d) Near UV CD spectrum. e) DCS analysis. f) Competition of anti‐CRM_197_ serum binding to the protein with CRM_197_ and its stapled form as inhibitors. g) Anti‐CRM_197_ IgG levels of CRM_197_ and CRM_197_‐**1** after first and second boost immunizations in mice, 2 weeks apart.

To evaluate the biological effects resulting from the introduction of the oxetane staple into CRM_197_, we first assayed the capacity of competing with the binding of anti‐CRM_197_ serum to the proteins. We found that the stapled CRM_197_‐**1** induces an inhibition that was slightly lower compared to the unmodified protein (Figure 4 f). In contrast, a much better inhibition of the binding to a commercial anti‐diphtheria toxoid human recombinant monoclonal antibody was observed for CRM_197_‐**1** compared to the unmodified protein (Supporting Information, Figure S33a). To ascertain that protein epitopes were not impaired by the chemical modification of the disulfide bond, groups of 8 BALB/c mice were immunized with both unmodified CRM_197_ and the stapled CRM_197_‐**1** (Figure 4 g). Remarkably, these in vivo experiments demonstrated that CRM_197_‐**1** induced a statistically significant higher level of anti‐protein antibodies respect to the unmodified protein. The antibodies generated by CRM_197_‐**1** had a threefold higher avidity for the protein antigen compared to the anti‐CRM_197_ serum (avidity index=0.8±0.4 m for CRM_197_‐**1** vs. 0.3±0.1 m for CRM_197_), as determined by ELISA using thiocyanate elution (Supporting Information, Figure S33b). These data, together with 200 ns MD simulations performed on both proteins (Supporting Information, Figure S12), suggest that the oxetane bridging of the disulfide bond does not cause relevant structural modifications on the protein but results in improved immunogenic activity in vivo, most likely through chemical stabilization of the antigen against proteases and/or other degradation factors.

In summary, we have presented an efficient method for oxetane stapling of Cys residues present as native disulfides on peptides and proteins under mild and biocompatible aqueous conditions. The four‐membered oxetane ring has an ideal distance to enable direct stapling of native disulfides on several protein scaffolds, including antibodies. This approach is however dependent on solvent accessibility of the disulfide within the protein of interest. Furthermore, and unlike current protocols, this method does not require prior sequence engineering neither purification after the disulfide reduction step. The selective installation of the oxetane motif enables stabilization of folded structures and results in disulfide‐grafted products with enhanced bioactivity that are stable under biological conditions. We demonstrate the value of oxetane graft installation on protein through the regioselective disulfide stapling of the protein carrier CRM_197_ that showed a significant increase in its immunogenicity in vivo. Because many therapeutic proteins feature Cys residues in the form of disulfide bonds, we anticipate that their direct modulation through oxetane grafting can, in principle, be used as a general strategy to enhance their in vivo stability and to fine‐tune their structure for optimal pharmacokinetics and activity.

## Conflict of interest

N.M.S., S.S., O.B., F.Corzana, and G.J.L.B. are listed as inventors on a pending patent application related to the technology described in this work. F.Carboni, D.O., and R.A. are employees of GSK companies.

## Supporting information

As a service to our authors and readers, this journal provides supporting information supplied by the authors. Such materials are peer reviewed and may be re‐organized for online delivery, but are not copy‐edited or typeset. Technical support issues arising from supporting information (other than missing files) should be addressed to the authors.

Supplementary
