# Supplementary material for: Oxetane Grafts Installed Site‐Selectively on Native Disulfides to Enhance Protein Stability and Activity In Vivo
Source: Angew Chem Int Ed Engl. 2017 Oct 20;56(47):14963–7. doi: 10.1002/anie.201708847 (PMC5698723; doi:10.1002/anie.201708847)
Supplement: Supplementary file 1 — Supplementary [file ANIE-56-14963-s001.pdf]

Angewandte Chemie
Eine Zeitschrift der Gesellschaft Deutscher Chemiker

Supporting Information

Oxetane Grafts Installed Site-Selectively on Native Disulfides to Enhance Protein Stability and Activity In Vivo

*Nuria Martínez-Sáez[+], Shuang Sun[+], Davide Oldrini, Pietro Sormanni, Omar Boutureira, Filippo Carboni, Ismael Compañón, Michael J. Deery, Michele Vendruscolo, Francisco Corzana, Roberto Adamo, and Gonçalo J. L. Bernardes**

anie_201708847_sm_miscellaneous_information.pdf

Supporting Information

Table of Contents

1. Reagents and general procedures 2

2. Peptide stapling 3

2.1 Linear peptide and peptide **CAAAC** 3

2.2 Cyclic peptides 5

2.3 Tryptophan fluorescence spectroscopy 7

3. Structural effects of macrocyclisation of **CAAAC** with **1**

3.1 2D NOESY experiments 8

3.2 Circular Dichroism (CD) spectroscopy 9

3.3 General Molecular Dynamics (MD) simulations protocol 9

3.4 Discussion 11

4. ϕ/ψ distribution obtained from 0.5 μs MD simulations for compounds **2-5** 13

5. MD simulations on proteins (Trx, stapled Trx-**1**, CRM_{197} and CRM_{197}-**1**) 15

6. Stability test of stapled cyclic peptides 17

7. General method for LC–MS 20

8. Thioredoxin (Trx) modification and characterization 21

9. Fab-Her modification and characterization 25

10. DesAb-$A\beta_{3-9}$ stapling and characterization 32

11. CRM_{197} stapling and activity studies 37

12. ^{1}H and ^{13}C NMR spectra of peptides 45

13. HPLC chromatograms of the peptides 52

14. References 54

1. Reagents and general procedures

Commercial reagents were used without further purification. Organic solvents used for reactions were of reagent grade. ^{1}H and ^{13}C NMR spectra were measured with a 500 MHz spectrometer with TMS as the internal standard. Multiplicities are quoted as singlet (s), doublet (d), doublet of doublets (dd), triplet (t), or multiplet (m). Spectra were assigned using COSY and HSQC. All NMR chemical shifts (δ) were recorded in ppm and coupling constants (J) were reported in Hz. Solid-phase syntheses were carried out in peptide synthesis reaction vessels (25 or 50 mL) with coarse porosity fritted glass support and Teflon stopcocks. Prep-scale reverse-phase chromatography was conducted with Agilent 1100 Series HPLC Value System [YMC-Pack Pro C18 column (5 μ, 250 mm × 10 mm)]. The eluent was acetonitrile (HPLC grade) with 0.1% trifluoroacetic acid and Millipore water with 0.1% trifluoroacetic acid. Somatostatine and Octeotride peptides were acquired in GeneCust Europe, Thioredoxin was bought from Sigma Aldrich, recombinant STTR2 was obtained in Origene Tecnologies, Fab-Her antibody was provided by Dr. Vijay Chudasama and CRM_{197} was provided by GSK vaccines.

2. Peptide stapling

2.1 Linear peptide

Synthesis of the linear peptide CAAAC

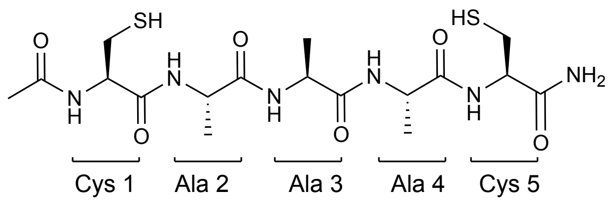

CAAAC

Pentapeptide CAAAC was prepared by standard manual Fmoc solid phase peptide synthesis using DIC/Oxyma pure[1] as coupling reagents and Rink Amide MBHA resin (0.1 mmol). The protected amino acids (4 equiv.) were assembly manually and activated with DIC (4 equiv.) and Oxyma pure (4 equiv.) in DMF (2 mL), using Fmoc-L-Ala-OH (337 mg, 0.4 mmol) and Fmoc-L-Cys(STrt)-OH (412 mg, 1 mmol) per coupling. The coupling reaction time was 45 min all cases. Fmoc deprotection was achieved in 30 min using piperidine/DMF (1:3, 2 mL). The *N*-terminus was acetylated with Ac_2O/pyridine (1:2) for 30 min. The cleavage step was carried out by treatment with TFA/TIS/H_2O/EDT (94:1:2.5:2.5) for 2 h. The crude peptide was precipitated with cold Et_2O. The purification was carried out by HPLC on an Agilent 1100 Series HPLC Value System [YMC-Pack Pro C18 column (5 μ, 250 mm × 10 mm)] at a flow rate of 3 mL/min, Grad: water+0.1% TFA / acetonitrile +0.1% TFA (95:5) → (61:39), 12 min, λ = 212nm, flow=3.5), R_t=10.3 min.

HRMS (ESI+) m/z: calcd. for $C_{17}H_{28}N_6NaO_6S_2[M+Na]^+$: 499.1404; found 499.1397.

^1H NMR (D_2O, 500 MHz) δ: 1.21 – 1.35 (m, 9H, $CH_{3\beta\ Ala}$), 1.95 (s, 3H, CH_3CO), 2.74- 2.88 (m, 4H, $CH_{2\beta\ Cys}$), 4.04 – 4.26 (m, 2H, $CH_{\alpha\ Ala}$), 4.29 – 4.40 (m, 2H, $CH_{\alpha\ Cys}$). ^1H NMR (H_2O/D_2O, 500 MHz) δ in ppm: 7.10 (s, 1H, NH_2), 7.46 (s, 1H, NH_2), 8.09 – 8.18 (m, 3H, NH_{Ala4}, NH_{Ala3}, NH_{Cys5}), 8.23 (d, *J* = 7.1 Hz, 1H, NH_{Cys1}), 8.39 (d, *J* = 5.8 Hz, 1H, NH_{Ala2}). ^{13}C NMR (D_2O, 126 MHz) δ: 16.2, 16.2, 16.3 (3 $C_{\beta\ Ala}$), 21.6 ($\underline{C}H_3CO$), 25.0, 25.1 ($2C_{\beta\ Cys}$), 49.6 , 49.7 , 49.8, 50.0 ($3C_{\alpha\ Ala}$), 55.2 , 55.3 ($C_{\alpha\ Cys}$), 171.9, 172.3 , 174.1 , 174.2 , 174.7 , 174.9 (6CO).

Synthesis of stapled peptide CAAAC-1

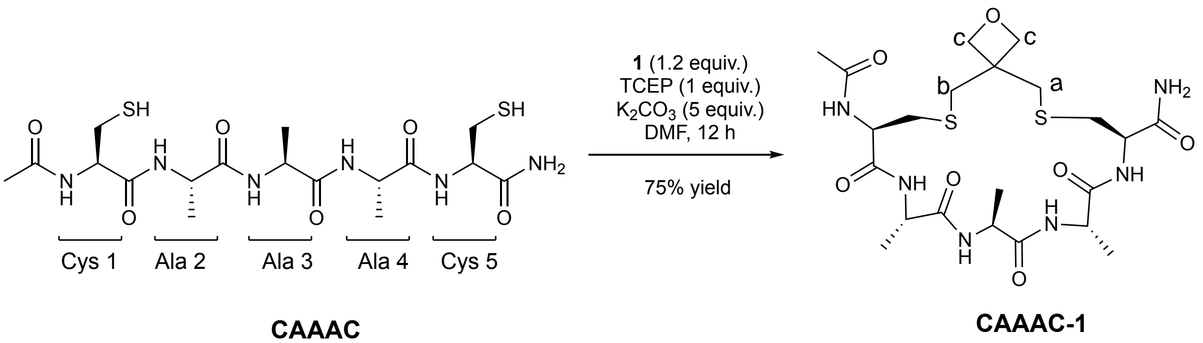

Peptide **2** (10 mg, 0.02 mmol) was dissolved in 10 mL of DMF. K_2CO_3 (14 mg, 0.10 mmol) and tris(2-carboxyethyl)phosphine (TCEP, 5.05 mg, 0.02 mmol) were then added. The solution was stirred for 1 h at rt. 3,3-bis(bromomethyl)oxetane **1** (3.4 µL, 0.025 mmol) was then added and stirred for additional 12 h. The crude peptide was purified by reversed-phase HPLC to obtain peptide **CAAAC-1** (5mg, 75% yield). YMC-Pack Pro C18 column (5 µ, 250 mm × 10 mm), Grad: water+0.1% TFA / acetonitrile +0.1% TFA (80:20) → (70:30), 30 min, λ = 212nm, flow=3.5), R_t=14.8 min.

HRMS (ESI+) m/z: calcd. for $C_{22}H_{36}N_6NaO_7S_2$ $[M+Na]^+$: 583.1979; found 583.1967.

^{1}H NMR (D_2O, 500 MHz) δ: 1.28 – 1.37 (m, 9H, $CH_{3\beta\ Ala}$), 1.95 (s, 3H, CH_3CO), 2.83 – 3.17 (m, 8H, $CH_{2\beta\ Cys}$, $2CH_{2a\ oxe}$), 4.10 – 4.28 (m, 3H, ($3C_{\alpha\ Ala}$), 4.37 – 4.53 (m, 6H, $C_{\alpha\ Cys}$, $2CH_{2c\ oxe}$). ^{1}H NMR (H_2O/D_2O, 500 MHz) δ in ppm: 7.16 (s, 1H, NH_2), 7.42 (s, 1H, NH_2), 7.83 (d, *J* = 6.3 Hz, 1H, NH_{Ala3}), 8.05 (m, 2H, NH_{Ala4}, NH_{Cys5}), 8.29 (d, *J* = 6.7 Hz, 1H, NH_{Cys1}), 8.55 (d, *J* = 6.0 Hz, 1H, NH_{Ala2}). ^{13}C NMR (D_2O, 126 MHz) δ: 15.7, 16.0, 16.9 (3 $C_{\beta\ Ala}$), 21.5 (OCH_3), 34.4, 34.3, 37.8, 38.4 (2 $2C_{\beta\ Cys}$, $2C_{a\ oxe}$), 43.3 ($C_{b\ oxe}$) 49.6, 50.1, 50.9 ($C_{\alpha\ Ala}$), 53.3, 53.7 ($C_{\alpha\ Cys}$), 79.9, 80.0 ($2C_{c\ oxe}$), 172.2, 172.4, 174.2, 174.5, 174.6, 175.1 (6CO).

2.2 Cyclic peptides

Synthesis of compound 3

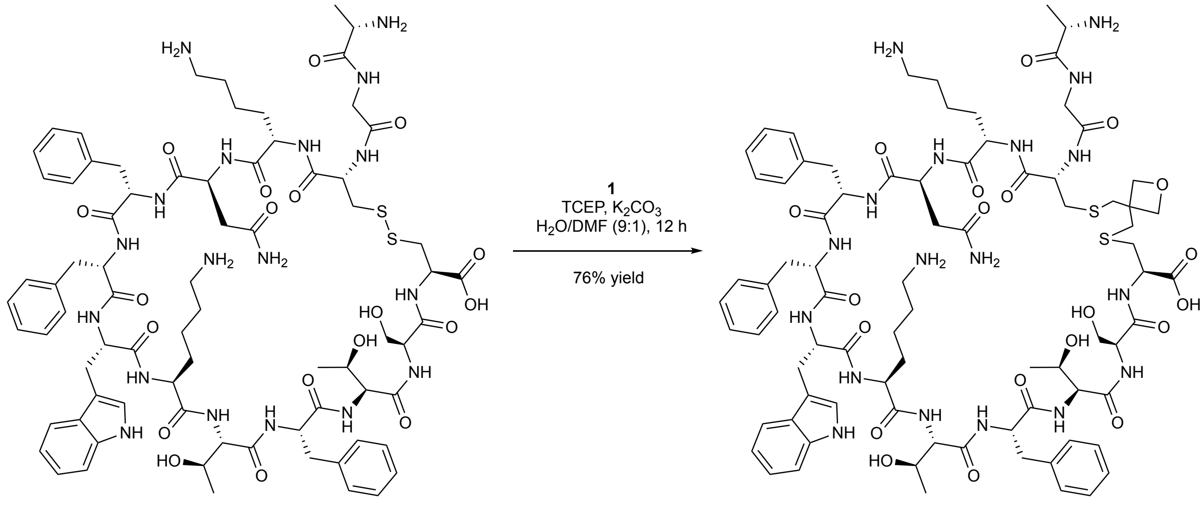

somatostatin 2 **3**

To a solution of natural somatostatin (10 mg, 0.006 mmol) in 10 mL H_2O, K_2CO_3 (4.0 mg, 0.030 mmol) and TCEP (1.5 mg, 0.006 mmol) were added. The solution was left to stir at rt for 1 h after which a 2 mg/mL solution of 3,3-bis(bromomethyl)oxetane **1** (1.2 µL, 0.007 mmol, 1.2 equiv.) in DMF was added to the mixture and left to stir for another 12 h. After completed, the reaction was purified to obtain peptide **3** with a 76% yield by reversed-phase HPLC and lyophilization. (YMC-Pack Pro C18 column (5 µ, 250 mm × 10 mm), isocratic: water+0.1% TFA / acetonitrile r+0.1% TFA (73-27), 15 min, λ = 212nm, flow=3.5), R_t= 11.1 min.

HRMS (ESI+) m/z: calcd. for $C_{81}H_{113}O_{20}N_{18}S_2$ $[M+H]^+$: 1721.7736; found 1721.7801.

Synthesis of compound **5**

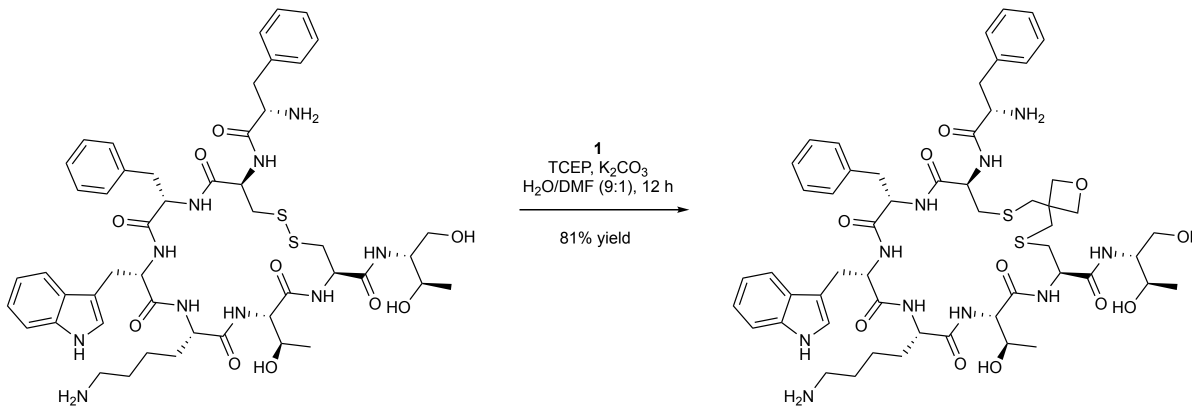

octreotide 4 **5**

To a solution of 0.1 mM of octreotide (10 mg, 0.009 mmol) in 10 mL H_2O was K_2CO_3 (6 mg, 0.049 mmol) and TCEP (2.3 mg, 0.009 mmol). The solution was left to stir at rt for 1 h after which a 2 mg/mL solution of 3,3-bis(bromomethyl)oxetane **1** (1.9 µL, 0.012 mmol, 1.2 equiv.) in DMF was added to the mixture and left to stir for another 12 h. After completed, the reaction was purified to obtain the stapled peptide **5** with an 81% yield by reversed-phase HPLC and lyophilisation. (YMC-Pack Pro C18 column (5 µ, 250 mm × 10 mm), Grad: water+0.1% TFA / acetonitrile +0.1% TFA (78:22) → (65:35), 13 min, λ = 212nm, flow=3.5), R_t=10.0 min. HRMS (ESI+) m/z: calcd. for $C_{54}H_{75}O_{11}N_{10}S_2$ $[M+H]^+$: 1103.5053; found 1103.5066.

2.3 Tryptophan fluorescence spectroscopy

Fluorescence spectroscopy was used to determine the dissociation constants of the cyclic peptides (somatostatin **2**, octreotide **3**, and the corresponding stapled peptides **3** and **5**) to somatostatin receptor 2 (SSTR2). All experiments were carried out in a Cary Eclipse spectrofluorometer (Varian) at 298K with SSTR2 at 1 μM, and concentrations of peptides varying from 0.1 to 7 μM in Tris·HCl buffer (25 mM, 150mM NaCl, pH 7.5). Fluorescence emission spectra were recorded in the 300–400 nm range with an excitation wavelength of 280 nm, with a slit width of 5 nm. The data analysis was performed considering a model with a single binding site.

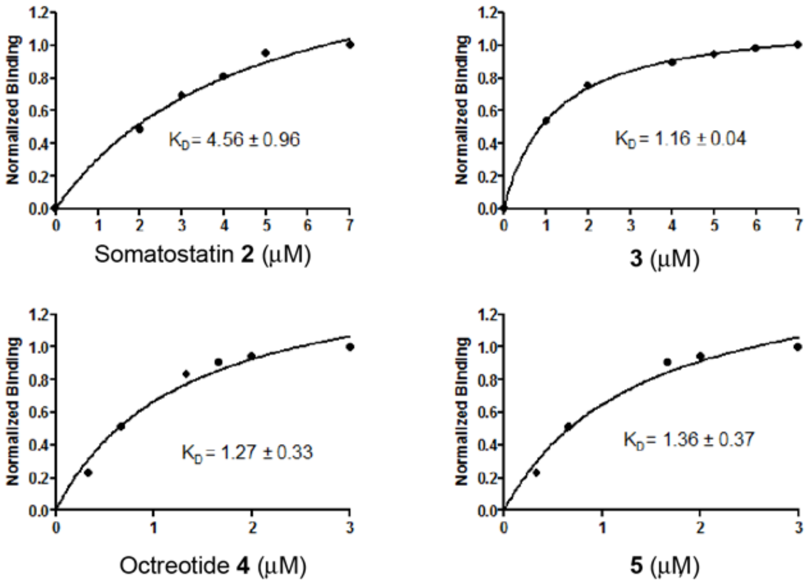

Figure S1. Binding affinity studies and K_D values (μM) obtained by tryptophan fluorescence spectrometry for somatostatin, octreotide and their stapled analogues **3** and **5** with somatostatin receptor 2 (SSTR2).

3 Structural effects of macrocyclisation of CAAAC with 1

3.1 2D NOESY experiments

NOESY experiments on peptides **CAAAC** and **CAAAC-1** were recorded on a Bruker Avance 500 spectrometer at 298 K and pH 6.5 in H_2O/D_2O (9:1). The experiments were conducted by using phase-sensitive ge-2D NOESY with WATERGATE for H_2O/D_2O (9:1) spectra. NOEs intensities were normalized with respect to the diagonal peak at zero mixing time. The number of scans used was 16 and the mixing time was 500 ms (Figure S2).

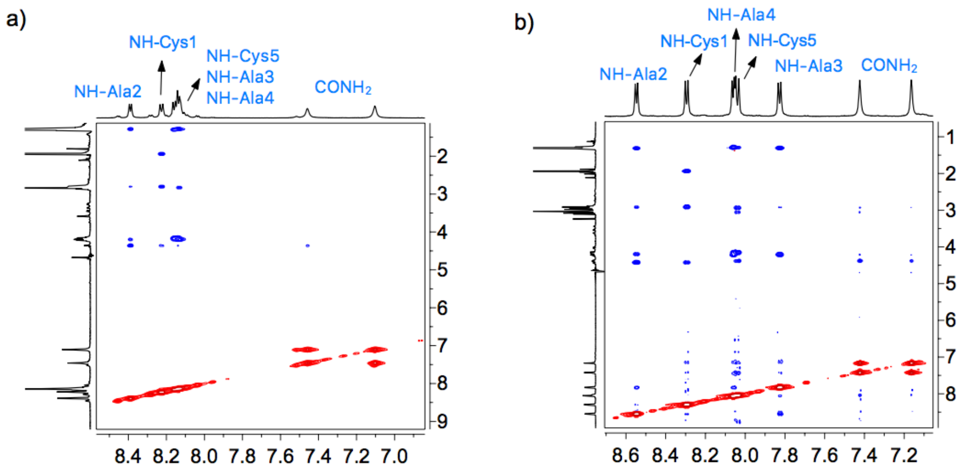

Figure S2. Section of the 500 ms NOESY spectra (500 MHz) of peptides **CAAAC** (a) and **CAAAC-1** (b) in H_2O/D_2O (9:1) at pH 6.5 and 20 ºC, showing amide–aliphatic cross-peaks. Diagonal peaks and exchange cross-peaks connecting NH protons and water are negative (red color). The NOE contacts are represented as positive cross-peaks (blue color).

3.2 Circular Dichroism (CD) spectroscopy. Solutions of peptides **CAAAC** and **CAAAC-1** were prepared from aqueous peptide stock solutions of accurate molecular concentrations determined by NMR. The final concentration of the peptide samples was 0.2 mg·mL^{-1} in a mixture 9:1 sodium phosphate buffer (10 mM, pH 7.2) and 2,2,2-trifluoroethanol (TFE). CD measurements were performed on an Aviv Model 410 spectrometer, which was routinely calibrated with (*1S*)-(+)-10-camphorsulfonic acid. Spectra were recorded at 298K with a 0.1 cm quartz cell over the wavelength range 250-189 nm at 50 nm·min^{-1}, with a bandwidth of 1.0 nm, the response time of 1 s, resolution step width of 1 nm and sensitivity of 20-50 Mdeg. Each spectrum represents the average of 5 scans. CD spectra were converted from raw ellipticity (θ, mdeg) to mean molar ellipticity per residue ([θ], deg·cm^2·dmol^{-1}·residue^{-1}).

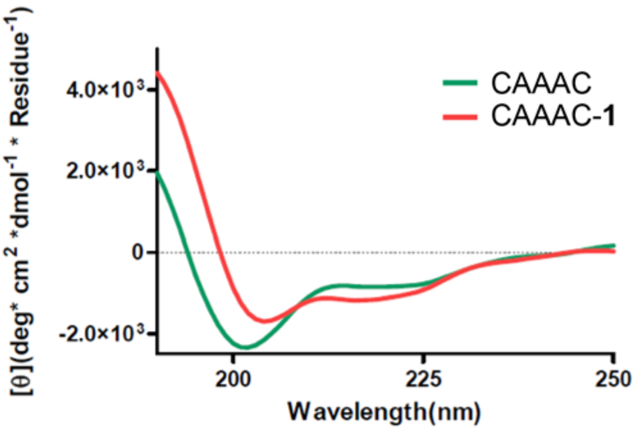

Figure S3. Circular dichroism spectra of free peptide **CAAAC** and stapled peptide **CAAAC-1** at 300 µM in a mixture 9:1 of Na_2HPO_4 buffer (10 mM, pH 7.2) and TFE.

3.3. General Molecular Dynamics simulations protocol

MD simulations were performed using Amber16.[2] Parameters for the unnatural residues were generated by the *antechamber* module using a combination of ff14SB[3] (for peptides **CAAAC** and **CAAAC-1** and proteins) or ff15ipq[4] (for somatostatin, octreotide and peptides **4** and **5**) and the general Amber force field (GAFF) parameters,[5] with partial charges set to fit the electrostatic potential generated with HF/6-31G(d) by RESP algorithm implemented in Amber.

The charges are calculated according to the Merz-Singh-Kollman scheme using Gaussian 09.[6] Each peptide (or protein) was immersed in a box with a 10 Å buffer of TIP3P[7] water molecules and neutralized by adding explicit counter ions (Na^+ or Cl^-). A two-stage geometry optimization approach was used. The first stage minimizes only the positions of solvent molecules and ions, and the second stage is an unrestrained minimization of all the atoms in the simulation cell. The systems were then gently heated by incrementing the temperature from 0 to 300 K under a constant pressure of 1 atm and periodic boundary conditions. Harmonic restraints of 30 kcal/mol were applied to the solute, and the Andersen temperature coupling scheme was used to control and equalize the temperature. The time step was kept at 1 fs during the heating stages. Water molecules are treated with the SHAKE algorithm such that the angle between the hydrogen atoms is kept fixed. Long-range electrostatic effects are modeled using the particle-Mesh-Ewald method.[8] An 8-Å cut-off was applied to Lennard-Jones interactions. Each system was equilibrated for 2 ns with a 2-fs time step at a constant volume and temperature of 300 K. Production trajectories were then run for additional 0.5 μs (for peptides) or 200 ns (for proteins) under the same simulation conditions.

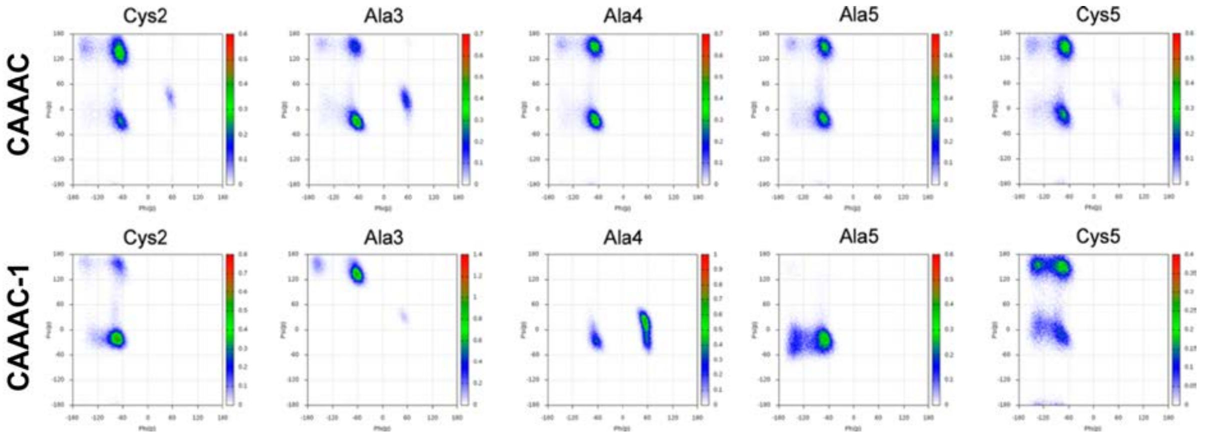

Figure S4. ϕ/ψ distributions obtained through 0.5 μs MD simulations in explicit water for the peptide backbone of peptides **CAAAC and** and **CAAAC-1**.

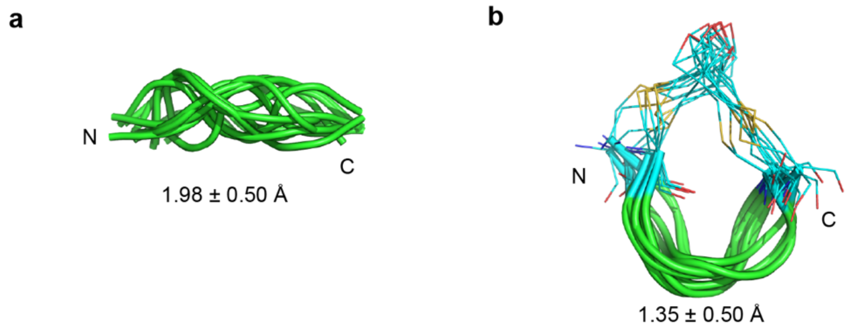

Figure S5. Structural ensembles obtained for peptide **CAAAC** (a) and stapled peptide CAAAC-**1** (b) through 0.5 μs MD simulations. The backbone is in green and carbon atoms of Cys residues as well as of the oxetane moiety are in cyan. The numbers indicate the root-mean-square deviation (RMSD) for heavy-atom superimposition of the backbone with respect to the average structure. N, *N*-terminus; C, *C*-terminus.

3.4 Discussion

Recent macrocyclization strategies based on aromatic systems[9] might disfavor the formation of stapled peptides due to the ring strain imposed by the aromatic moiety. Instead, the use of an oxetane, that "mimics" a more moldable propyl group, should permit access and accommodation of well-defined three-dimensional structures. To verify this hypothesis, we used a combination of NMR and molecular dynamics (MD) simulations to determine the structural changes induced by the incorporation of the oxetane into peptide **CAAAC**. Inspection of the 2D-NOESY spectra showed substantial differences between the unstapled and stapled peptides in terms of their conformational preferences (**Figure S2**). While clear medium-size NOE cross-peaks were observed in the amide region for peptide **CAAAC-1**, which are characteristic of a main folded conformation in solution, the absence of these NOE cross-peaks in unstapled peptide **CAAAC** is in agreement with an extended disposition of the backbone.[10] Both, the CD spectra (**Figure S3**) and structural ensembles obtained through 0.5 μs MD simulations performed on these peptides are in line with these observations (**Figure**

S4). According to these calculations, peptide **CAAAC** is quite flexible in solution, presenting a random-coil distribution for the peptide backbone. Conversely, stapled peptide **CAAAC-1** is rigid, showing a folded backbone promoted by the incorporation of the four-membered ring oxetane scaffold (**Figure S5**). The average distance S–S in **CAAAC-1** was calculated to be 4.3±0.3 Å, which is twice the value observed for a conventional disulfide bond.

4 ϕ/ψ distribution obtained from 0.5 μs MD simulations for compounds 2-5

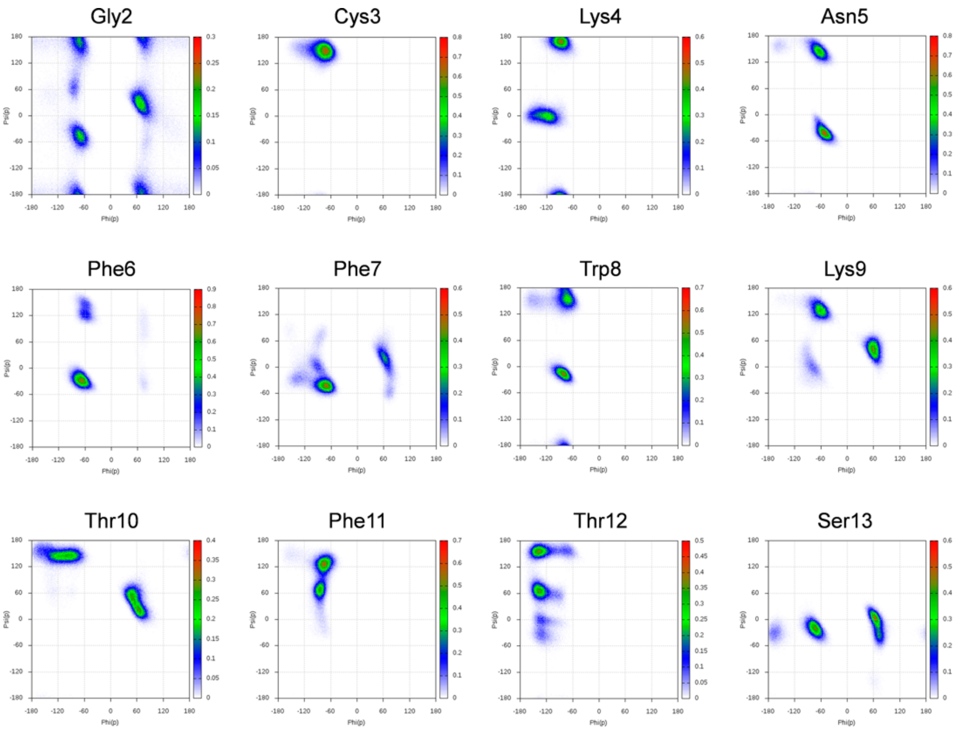

Figure S6. ϕ/ψ distributions obtained through 0.5 μs MD simulations in explicit water for the peptide backbone of somatostatin **2**.

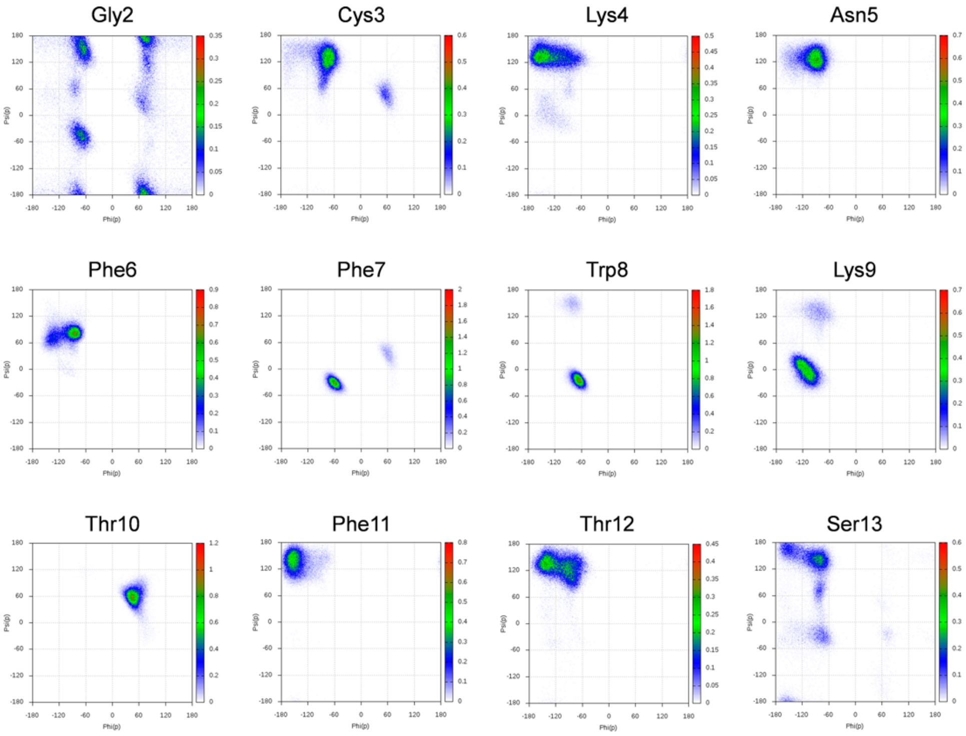

Figure S7. ϕ/ψ distributions obtained through 0.5 μs MD simulations in explicit water for the peptide backbone of stapled somatostatin **3**.

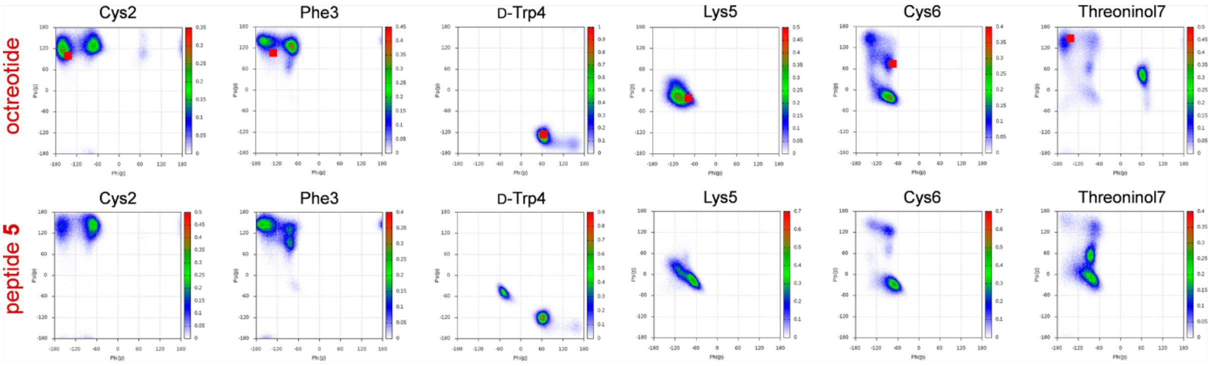

Figure S8. ϕ/ψ distributions obtained through 0.5 μs MD simulations in explicit water for the peptide backbone of octreotide **4** and peptide **5**. The average structure of octreotide previously reported in DMSO is shown in red.[11]

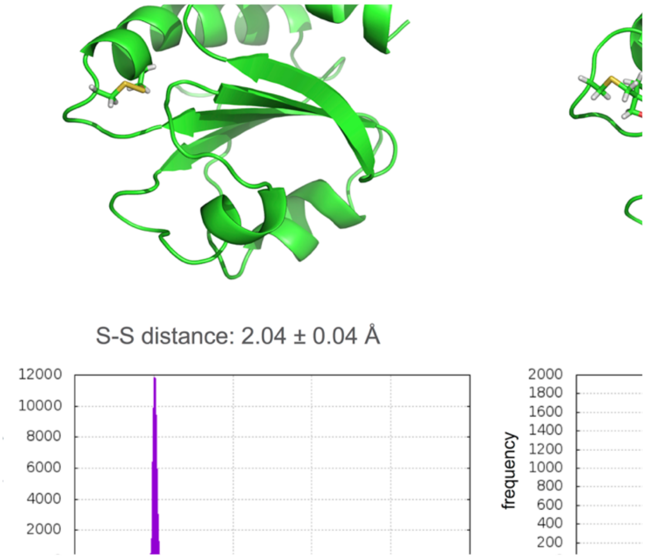

Figure S9. Distribution of S-S distance obtained through 200 ns MD simulations in explicit solvent for Trx (A) and the stapled Trx–**1** (B) Pdb ID: 2TRX.

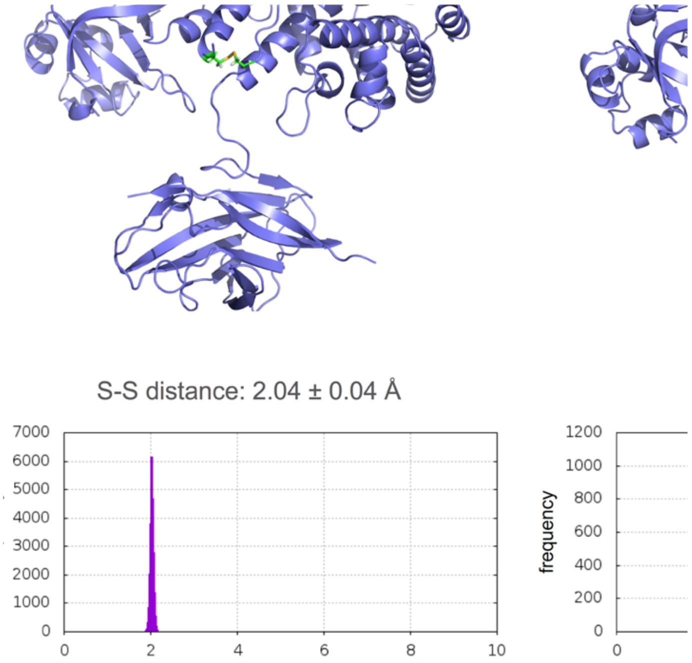

Figure S10. Distribution of S-S distance obtained through 200 ns MD simulations in explicit water for CRM_{197} (A) and the stapled CRM_{197}-**1** (B) (Pdb ID: 4AE0).

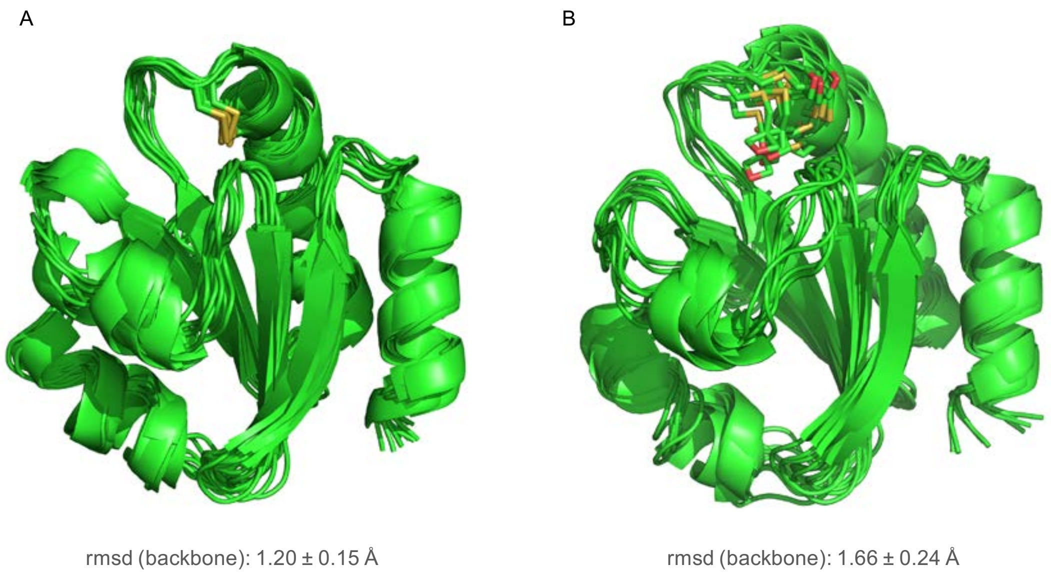

Figure S11. Structural ensembles obtained through 200 ns MD simulations for Trx (A) and Trx-**1** (B). The numbers indicate the root-mean-square deviation (RMSD) for superimposition of the backbone with respect to the starting structure (Pdb ID: 2TRX).

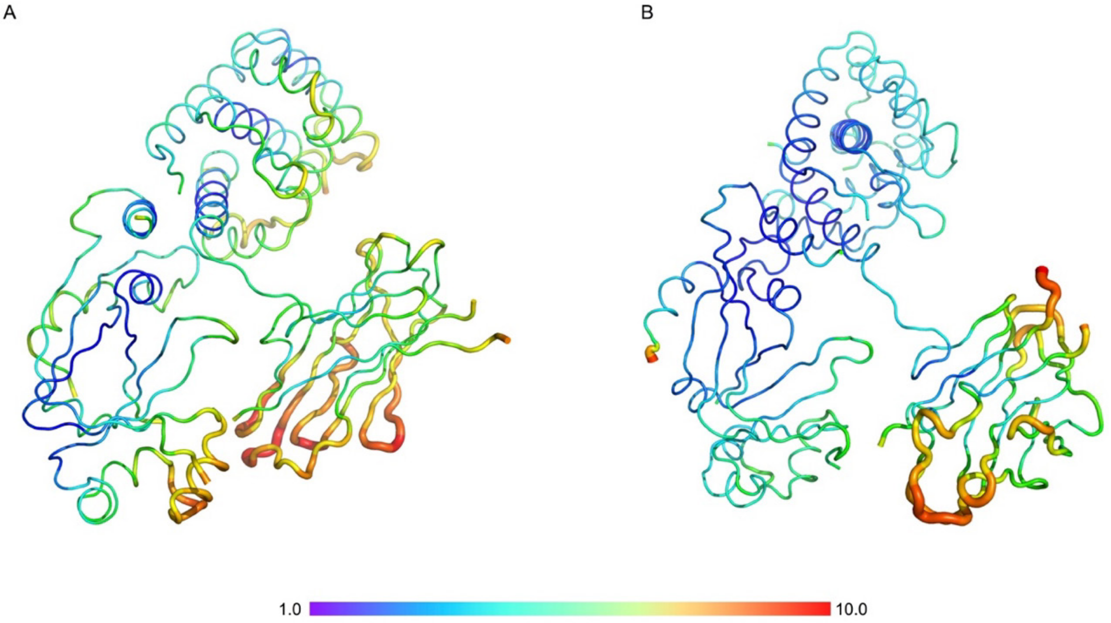

Figure S12. Atomic fluctuation (Cα) analysis (in Å) for CRM_{197} (A) and stapled CRM_{197}-**1** (B) obtained through 200 ns MD simulations. The data presented corresponds to the average structure throughout the simulations.

6. Stability test of stapled cyclic peptides

Stability of oxetane stapled Octreotide/Somatostatain in GSH solution

A 100 μL aliquot of stapled peptide (around 1 mM) in H_2O was treated with 5.3 μL of a 20 mM GSH solution (to 1 mM) and the resulting mixture vortexed for 30 s and then shaken at 37 ^{o}C. After 48 h, an aliquot of each reaction mixture was analysed by HPLC [YMC-Pack Pro C18 column (5 μ, 250 mm × 10 mm), isocratic: acetonitrile+0.1% TFA /water+0.1% TFA (73-27), 15 min, λ = 212nm, flow=3.5)]. No significant degradation of stapled peptide was observed at 48 h.

Note: the Rt value of the stapled octreotide ***5*** *varies slightly from that reported in the synthesis section due to the different method employed in the HPLC purification.*

Stability of oxetane stapled Octreotide/Somatostatain in human plasma

A 100 μL aliquot of stapled peptide (around 1 mM) in H_2O was treated with 1 μL of reconstituted human plasma (Sigma-Aldrich) and the resulting mixture vortexed for 30 s and then shaken at 37 ^{o}C. After 48 h, an aliquot of each reaction mixture was analysed by HPLC (YMC-Pack Pro C18 column (5 μ, 250 mm × 10 mm), isocratic: acetonitrile+0.1% TFA /water+0.1% TFA (73-27), 15 min, λ = 212nm, flow=3.5)). No significant degradation of stapled peptide was observed at 48 h.

Note: the Rt value of the stapled octreotide ***5*** *varies slightly from that reported in the synthesis section due to the different method employed in the HPLC purification.*

6.1 Oxetane stapled somatostatin chromatograms

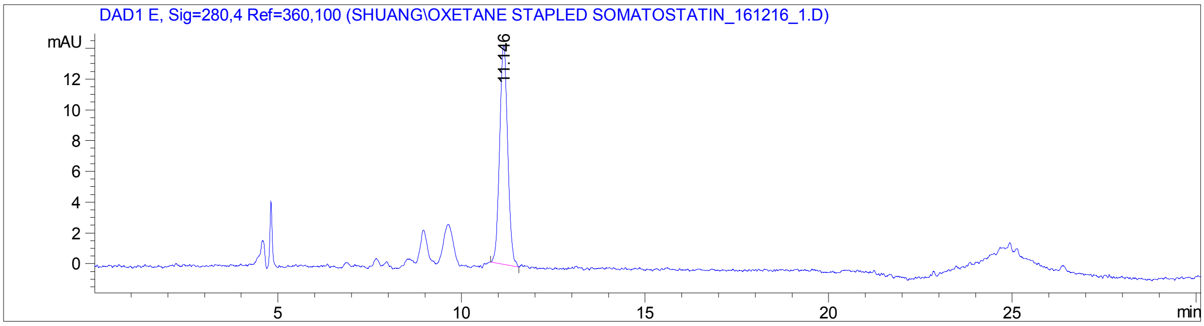

Figure S13. HPLC chromatogram of stapled somatostatin **3** before the stability tests.

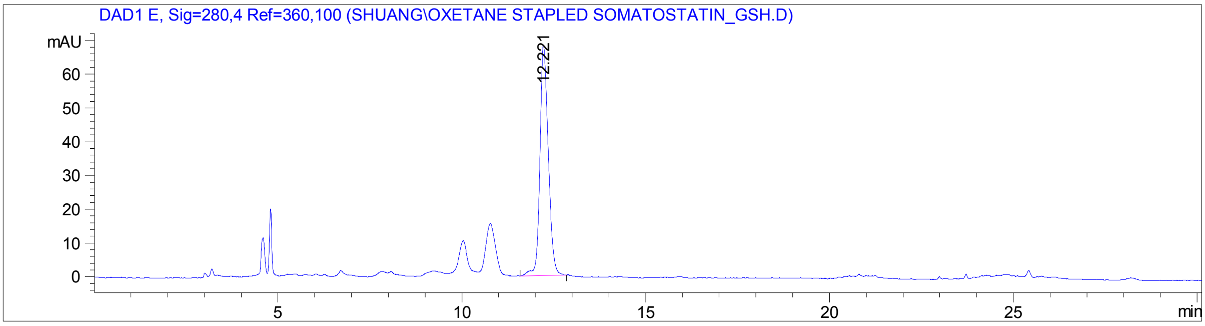

Figure S14. HPLC chromatogram of stapled somatostatin **3** in GSH solution.

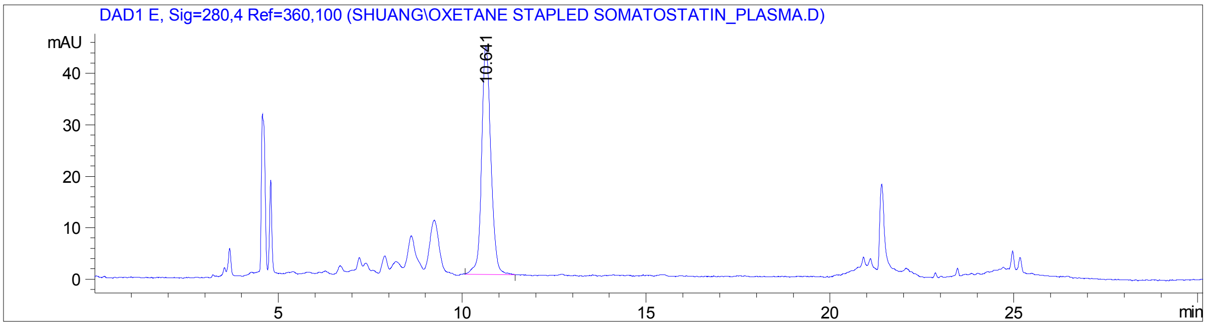

Figure S15. HPLC chromatogram of stapled somatostatin **3** in human plasma.

6.2 Oxetane Stapled Octreotide Chromatograms

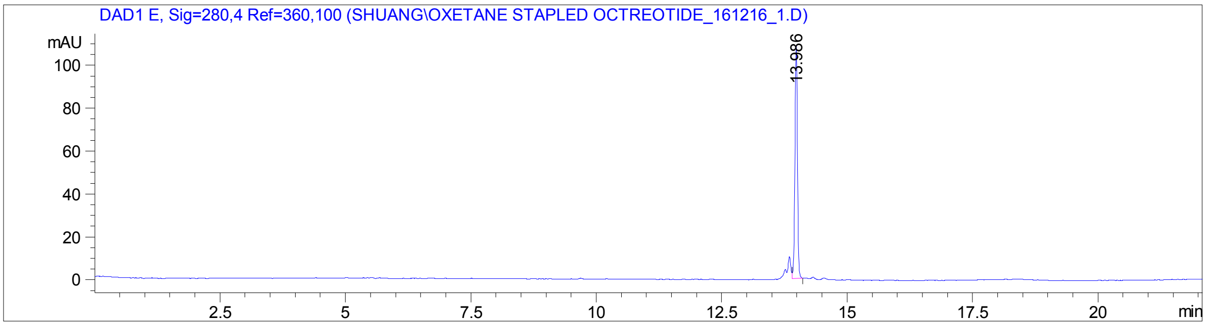

Figure S16. HPLC chromatogram of stapled octreotide **5** before the stability tests.

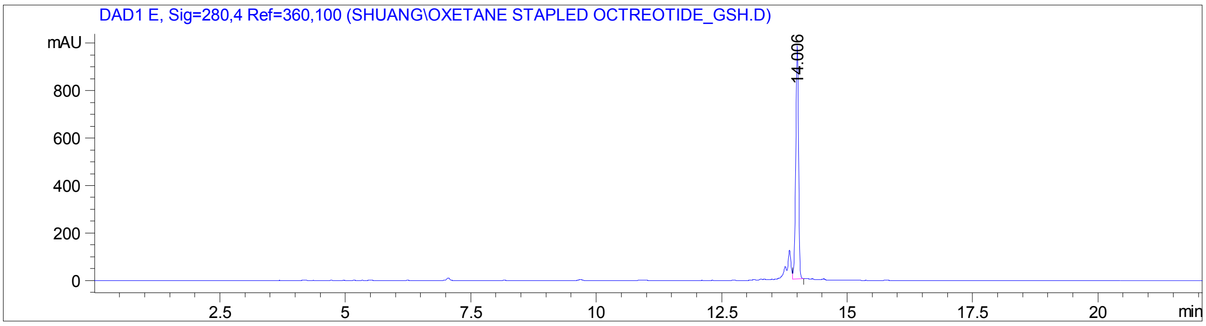

Figure S17. HPLC chromatogram of stapled octreotide **5** in GSH solution.

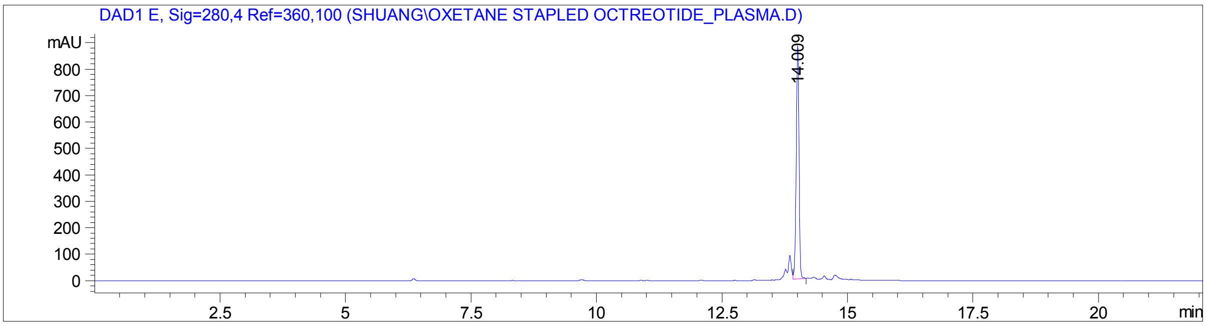

Figure S18. HPLC chromatogram of stapled octreotide **5** in human plasma.

7. General method for LC–MS

Liquid chromatography-mass spectrometry (LC–MS) was performed on a Xevo G2-S TOF mass spectrometer coupled to an Acquity UPLC system using an Acquity UPLC BEH300 C4 column (1.7 μm, 2.1% 50 mm). Solvents A (water with 0.1% formic acid) and B (71% acetonitrile, 29% water, and 0.1% formic acid) were used as the mobile phase at a flow rate of 0.2 $mL\cdot min^{-1}$. The gradient was programmed as follows: 72% A to 100% B for 25 min; then 100% B for 2 min and after that 72% A for 18 min. The electrospray source was operated with a capillary voltage of 2.0 kV and a cone voltage of 40 V. Nitrogen was used as the desolvation gas at a total flow of 850 $L\cdot h^{-1}$. Total mass spectra were reconstructed from the ion series using the MaxEnt algorithm preinstalled on MassLynx software (v. 4.1 from Waters) according to the manufacturer's instructions. To obtain the ion series described, the major peak(s) of the chromatogram were selected for integration and further analysis.

8. Thioredoxin (Trx) modification and characterization

8.1 Preparation of the stapled Trx–1

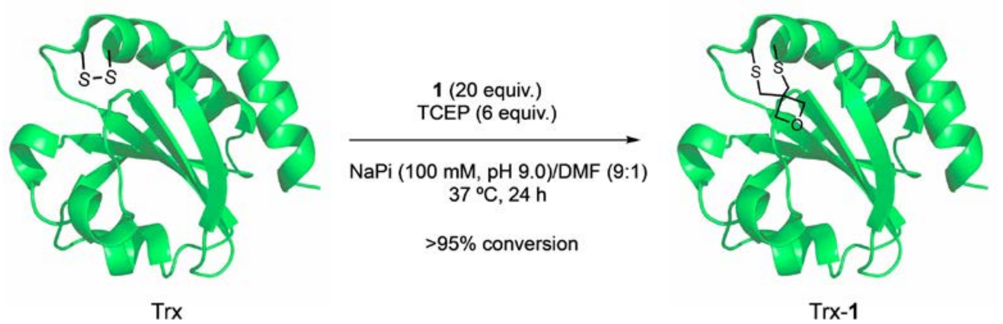

To a 0.5 mL eppendorf containing Trx (0.20 mg, 17 nmol) dissolved in sodium phosphate (NaP_i) buffer (100 mM, pH 9.2, 150 µL), was added TCEP (100 µL, 1 µmol/mL, 6 equiv.). The reaction was stirred at rtfor 2 h. A pre-mixed solution of 3,3-bis(bromomethyl)oxetane **1** in DMF (34 µL, 10 µmol/mL, 20 equiv.) was added and stirred for 24 h at 37 ºC. A 2 µL aliquot diluted with 90 µL of the same buffer was analysed directly by LC–MS and complete conversion was observed (see **Figures S19** and **S20**). Small molecules were removed from the reaction mixture by loading the sample onto a Zeba Spin Desalting Column previously equilibrated with NaP_i buffer (50 mM, pH 9.2). The sample was eluted via centrifugation (2 min, 1000xg). The protein solution was then flash frozen in liquid nitrogen and stored at –20 ºC.

Thioredoxin sequence (modified residues are in bold and underlined)

SDKIIHLTDDSFDTDVLKADGAILVDFWAEW**C**GP**C**KMIAPILDEIADEYQGKLTVAK

LNIDQNPGTAPKYGIRGIPTLLLFKNGEVAATKVGALSKGQLKEFLDANLA.

Isotopically Averaged Molecular Weight = 11675 Da

A typical analysis of a conjugation reaction by LC–MS is described below. The total ion chromatogram, combined ion series and deconvoluted spectra are shown for the starting material and product of the reaction of Trx with **1**. Identical analyses were carried out for all the conjugation reactions performed in this work.

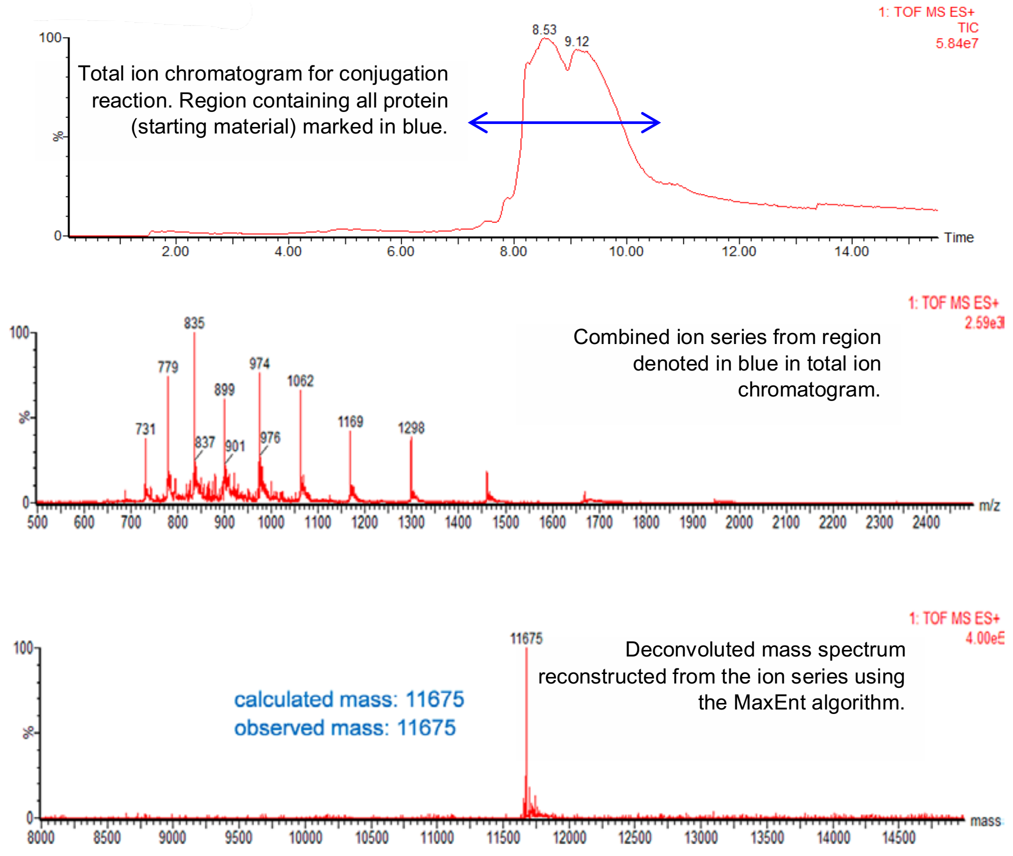

Figure S19. Chromatogram, combined ion series and deconvoluted mass spectra of Trx.

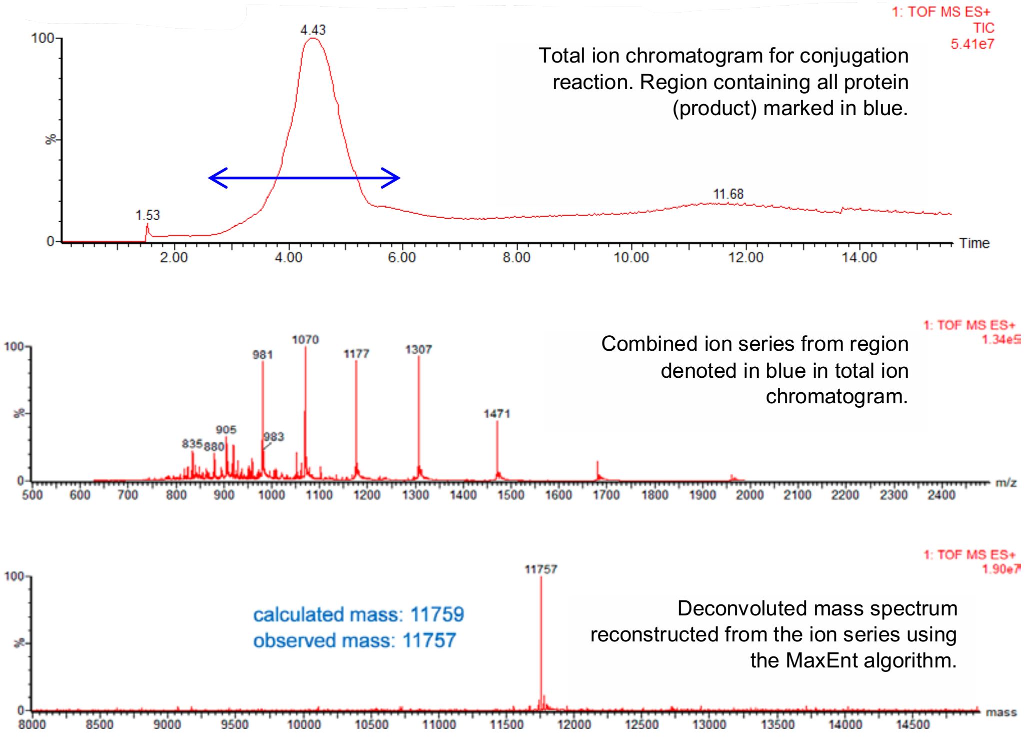

Figure S20. Chromatogram, combined ion series and deconvoluted mass spectra of the Trx–**1**.

8.2 Bioactivity measurement of Trx and Trx–1

Trx and Trx–**1** activity were measured by following the reduction of insulin previously described.[12]

The standard Trx (or Trx–**1**) assay mixture, prepared in 200 µL overall volume, contained sodium phosphate buffer (50 mM, pH 7.0), 1 mM EDTA, 0.15 mM human insulin and 1 mM dithiothreitol (Figure S21). The amounts of Trx or Trx–**1** were varied and the concentrations of the protein were determined by Bradford assay. Samples were run in duplicate and the increase in turbidity from the reduction of insulin was monitored at 650 nm at 30 ºC by a Tecan plate reader. The kinetic curves were corrected by subtracting from insulin reduction by dithiothreitol alone. The corrected slopes from the kinetic data (ΔmAU/min), in the linear region, were plotted as a function of the concentration of protein.

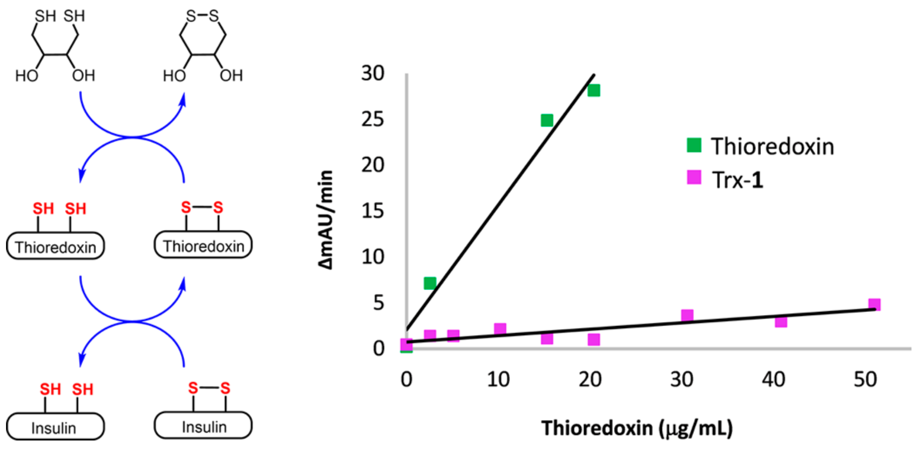

Figure S21. Bioactivity measurement of Trx and stapled Trx–**1**.

8.3 CD spectra of Thioredoxin and Trx-1

CD was carried out following the above-mentioned methodology (section 3). The final concentration of the protein samples was 6 μM in sodium phosphate buffer (10 mM, pH 7.2).

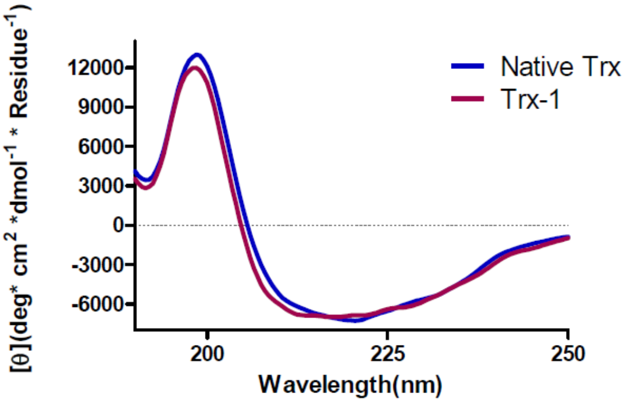

Figure S22. Circular dichroism spectra of Thioredoxin and the stapled Thioredoxin (Trx-**1**) in Na_2HPO_4 buffer (10 mM, pH 7.2).

9. Fab-Her modification and characterization

9.1 Preparation of the stapled Fab-Her (Fab-Her–1)

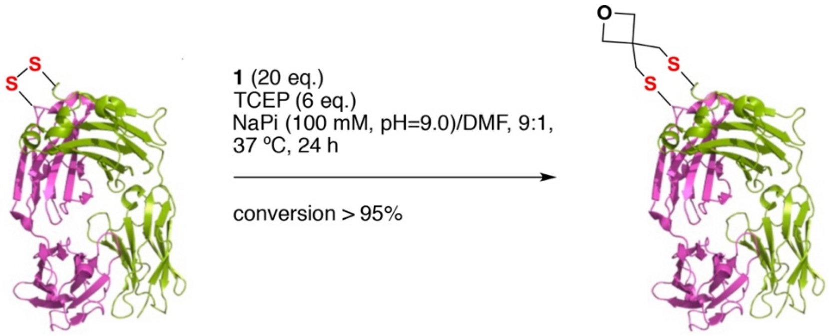

To a 0.5 mL eppendorf containing the Fab-Her fragment (0.005 µmol) dissolved in NaP_i buffer (100 mM, pH 9.0, 120 µL), was added TCEP (30 µL, 1 µmol/mL, 6 equiv.). The reaction was stirred at room temperature for 2 h. A pre-mixed solution of oxetane **1** in DMF (10 µL, 10 µmol/mL, 20 equiv.) was added and stirred for 24 h. A 2 µL aliquot diluted with 90 µL of the same buffer was analysed directly by LC–MS and full conversion was observed (see **Figures S23** and **S24**). Small molecules were removed from the reaction mixture by loading the sample onto a Zeba Spin Desalting Column previously equilibrated with NaPi buffer (50 mM, pH 9.2). The sample was eluted via centrifugation (2 min, 1000xg). The protein solution was then flash frozen in liquid nitrogen and stored at –20 ºC.

Fab-Her sequence (modified residue is in bold and underlined):

Light chain:

DIQMTQSPSSLSASVGDRVTITCRASQDVNTAVAWYQQKPGKAPKLLIYSASFLYSG

VPSRFSGSRSGTDFTLTISSLQPEDFATYYCQQHYTTPPTFGQGTKVEIKRTVAAPSVF

IFPPSDEQLKSGTASVVCLLNNFYPREAKVQWKVDNALQSGNSQESVTEQDSKDSTY

SLSSTLTLSKADYEKHKVYACEVTHQGLSSPVTKSFNRGE**C**

Heavy chain:

EVQLVESGGGLVQPGGSLRLSCAASGFNIKDTYIHWVRQAPGKGLEWVARIYPTNG

YTRYADSVKGRFTISADTSKNTAYLQMNSLRAEDTAVYYCSRWGGDGFYAMDYW

GQGTLVTVSSASTKGPSVFPLAPSSKSTSGGTAALGCLVKDYFPEPVTVSWNSGALT

SGVHTFPAVLQSSGLYSLSSVVTVPSSSLGTQTYICNVNHKPSNTKVDKKVEPPKS**C**D

KTH

Isotopically Averaged Molecular Weight = 47638 Da

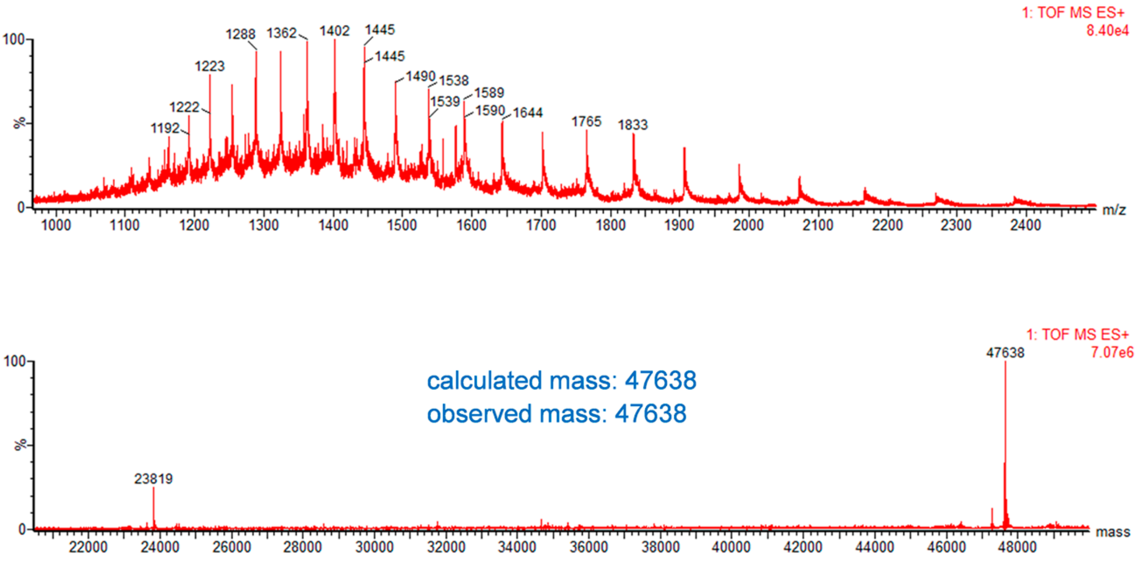

Figure S23. Combined ion series and deconvoluted mass spectrum of Fab-Her.

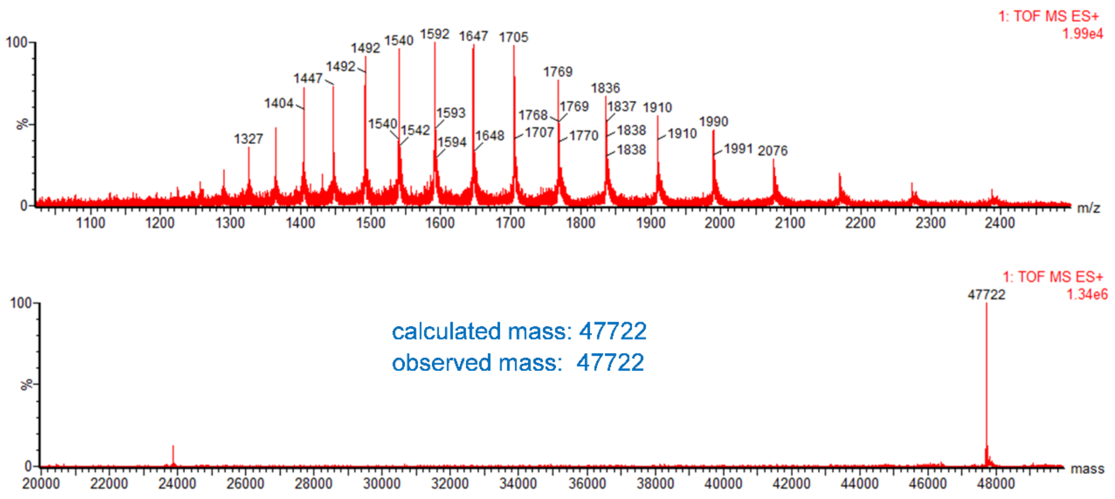

Figure S24. Combined ion series and deconvoluted mass spectrum of reaction of Fab-Her with **1** after 24 h at 37 ºC.

9.2 Stability of Fab-Her–1

Stability of the modified Fab-Her–1 fragment in human plasma

A 10 µL aliquot of the stapled Fab-Her–**1** (33 µM) in NaP_i buffer (50 mM, pH 9.2) was treated with 1 µL of reconstituted human plasma (Sigma-Aldrich) at room temperature and the resulting mixture vortexed for 30 s and then shaken at 37 ºC overnight. After 24 h, a 2.5 µL aliquot of each reaction mixture was analysed by LC–MS (2.5 µL aliquot diluted with 8 µL NaP_i buffer (50 mM, pH 9.2)). No detectable degradation of Fab-Her–**1** was observed at 24 h (**Figure S25**).

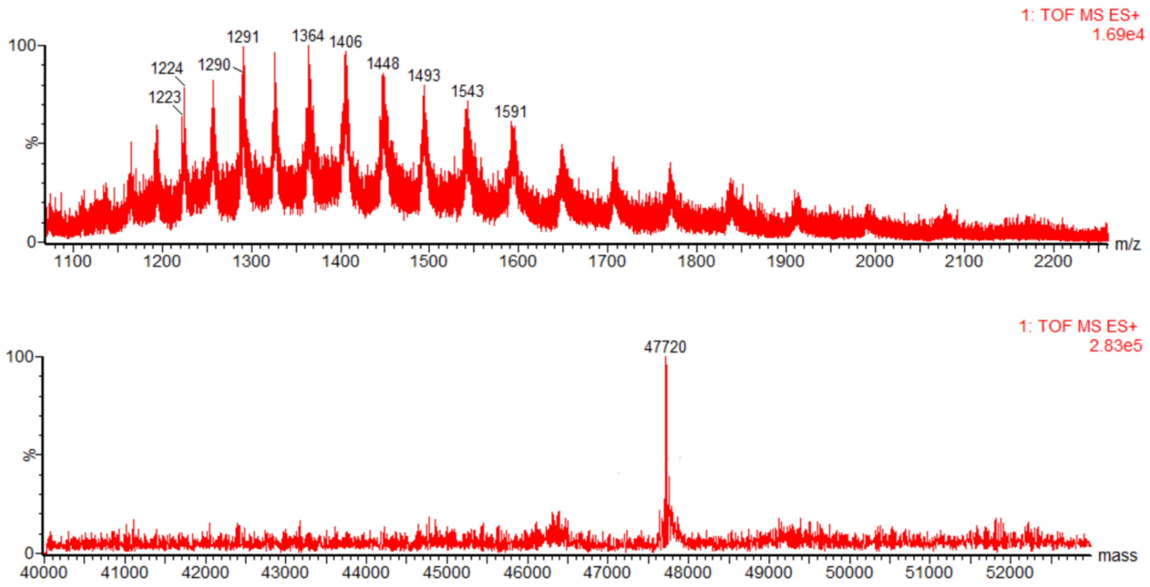

Figure S25. Combined ion series and deconvoluted mass spectrum after incubation of stapled Fab-Her–**1** with human plasma for 24 h at 37 ºC.

*Stability of the modified Fab-Her-**1** in the presence of glutathione (GSH)*

A 10 µL aliquot of Fab-Her–**1** (33 µM) in NaPi buffer (50 mM, pH 9.2) was treated with 1 µL of a 20 mM GSH solution (6 mg glutathione dissolved in 1 mL of 50 mM NaPi buffer at pH 9.2) at rt and the resulting mixture vortexed for 30 s and then shaken at 37 ºC overnight. After 24 h, a 2.5µL aliquot of each reaction mixture was analysed by LC–MS (2.5 µL aliquot diluted with 8 µL of 50 mM NaPi buffer at pH 9.2). No significant degradation of Fab-Her-**1** was observed at 24 h (Figure S26).

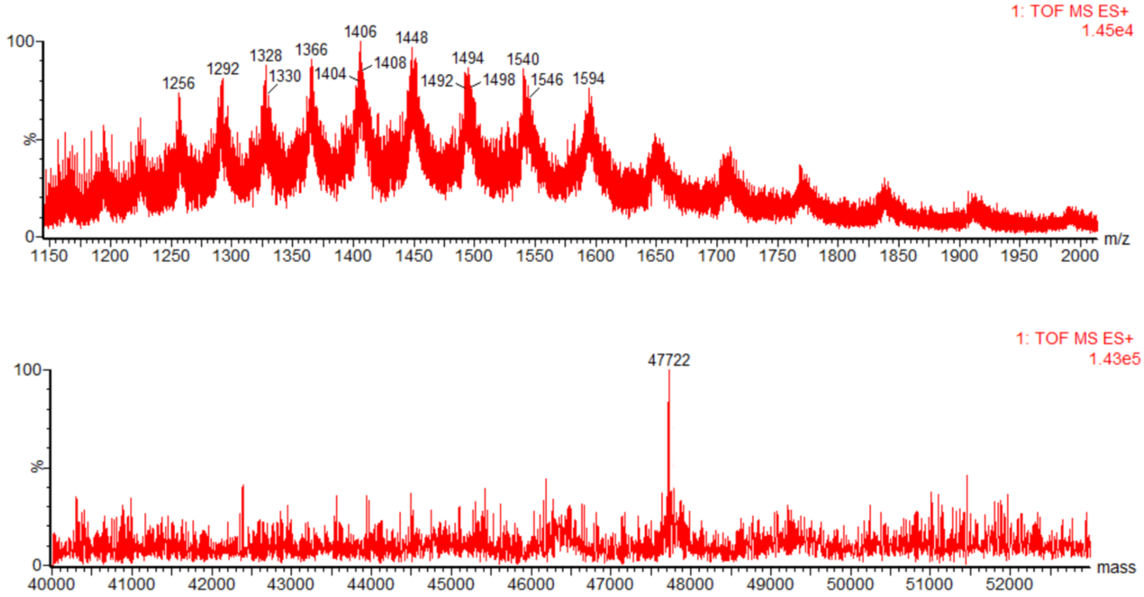

Figure S26. Combined ion series and deconvoluted mass spectrum after incubation of stapled Fab-Her–**1** with GSH (1 mM) for 24 h at 37 ºC.

9.3 Determination of the binding affinity of Fab-Her–1

Biotinylation of Antibodies

Non-modified Fab-Her fragment and modified Fab-Her-**1** were conjugated to a biotin linker using Biotin-$(PEG)_4$-NHS (Thermofisher Scientific) to carry out BLI experiments using Streptavidin (SA) Biosensors. A solution of EZ-Link NHS- (PEG)4-Biotin (20 µL, 200 µM in PBS) was added to the corresponding protein (20 µL, 20 µM in PBS) and was left at room temperature for 30 minutes. The crude reaction mixture was buffer exchanged with PBS for 3 times to remove the excess of NHS-$(PEG)_4$-Biotin, obtaining a biotin-to-antibody ratio around 1.5.

Bio-layer Interferometry (BLI)

Binding assays were performed on an Octet Red Instrument (fortéBIO). Ligand immobilization, binding reactions, regeneration, and washes were conducted in wells of black polypropylene 96-well microplates. Non-modified Fab-Her fragment and modified Fab-Her-**1** (20 nM) were immobilized on Streptavidin (SA) Biosensors in PBS with 0.1% BSA and 0.02% tween at 30 ºC. Binding analysis was carried out at 25 ºC, 1000 rpm in PBS with 0.1% BSA and 0.02% tween, with a 600 s of association followed by a 2200 s of dissociation, using different concentrations of the recombinant HER2 receptor to obtain the association curve. Glycine pH 2.0 was used as a regeneration buffer. Data were analyzed using Data Analysis (fortéBIO), with Savitzky-Golay filtering (Figure S27). Binding was fitted to a 2:1 Heterogeneous ligand model, steady state analysis was performed to obtain the binding kinetics constants (K_D).

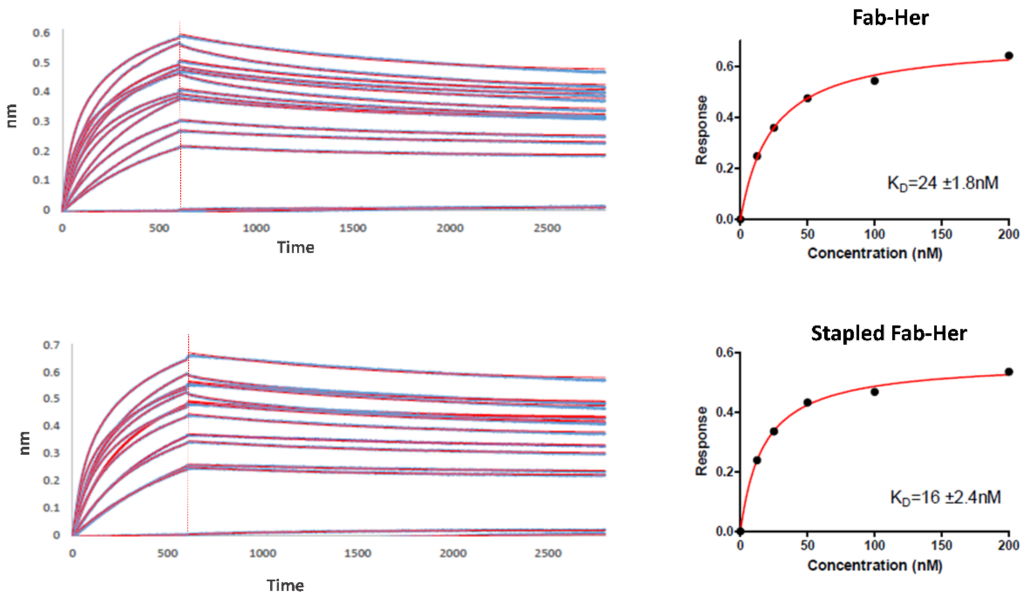

Figure S27. Bio-Layer Interferometry (BLI) curves (in blue) and fitting curves (in red) obtained for Fab-Her and Fab-Her–**1** fragments with Her2 receptor, together with the K_D constants derived from BLI experiments.

10. DesAb-A$\beta_{3\text{-}9}$ stapling and characterization

10.1 Antibody expression and purification

The designed antibody (DesAb) construct was expressed and purified from pRSET-b vector in *E. coli* BL21 (DE3) strain (Agilent Technologies). Cells were grown for about 5 h at 37 ºC and then overnight at 28 ºC into Overnight Express Instant TB Medium (Merck Millipore) supplemented with ampicillin (100 µg/mL). Cells were then harvested by centrifugation, resuspended in phosphate-buffered saline (PBS) with the addition of one EDTA-Free Complete Protease Inhibitor Mixture tablet (Roche), and lysed using sonication; cell debris were removed using centrifugation at 16,000 rpm (JA-25.50 Rotor; Beckman Coulter). The cleared lysate was loaded onto a Ni2+-NTA Superflow column (Qiagen), previously equilibrated with PBS. After washing with PBS containing 20 mM imidazole, the His-tagged DesAb was eluted with PBS containing 200 mM imidazole. Finally, a size exclusion chromatography (SEC) was carried out on the eluted fractions using a superdex 75 16/600 (GE Healthcare) column equilibrated in 20 mM sodium phosphate buffer (pH 8), 200 mM EDTA, and the peak corresponding to the monomeric DesAb was collected for experiments. Protein purity always exceeded 98% as determined by SDS/PAGE analysis.

10.2 Preparation of stapled DesAb-A$\beta_{3\text{-}9}$-1

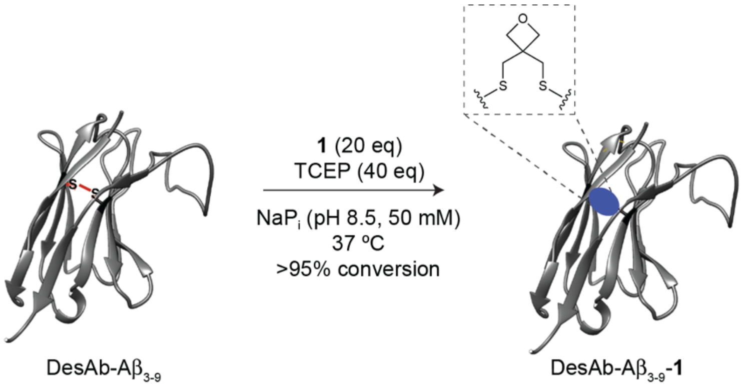

To a 0.5 mL Eppendorf containing designed antibody (25 µL, 120 µmol, 3 nmol) dissolved in sodium phosphate (NaPi) buffer (50 mM, pH 8.5, 150 µL), was added TCEP (12 µL, 10 mM, 40 equiv.). The reaction was stirred at 37 °C overnight. A pre-mixed solution of 3,3-bis(bromomethyl)oxetane **1** in DMF (6 µL, 10 mM, 20 equiv.) was added and stirred at 37 ºC for 3 days. A 10 µL aliquot analysed directly by LC–MS and complete conversion was observed (calculated mass, 19905 Da (native antibody, 19821 Da + oxetane staple, 84 Da); observed mass, 19904). Small molecules were removed from the reaction mixture by loading the sample onto a Zeba Spin Desalting Column previously equilibrated with 1X PBS pH 7.4. The sample was eluted via centrifugation (2 min, 1000 xg).

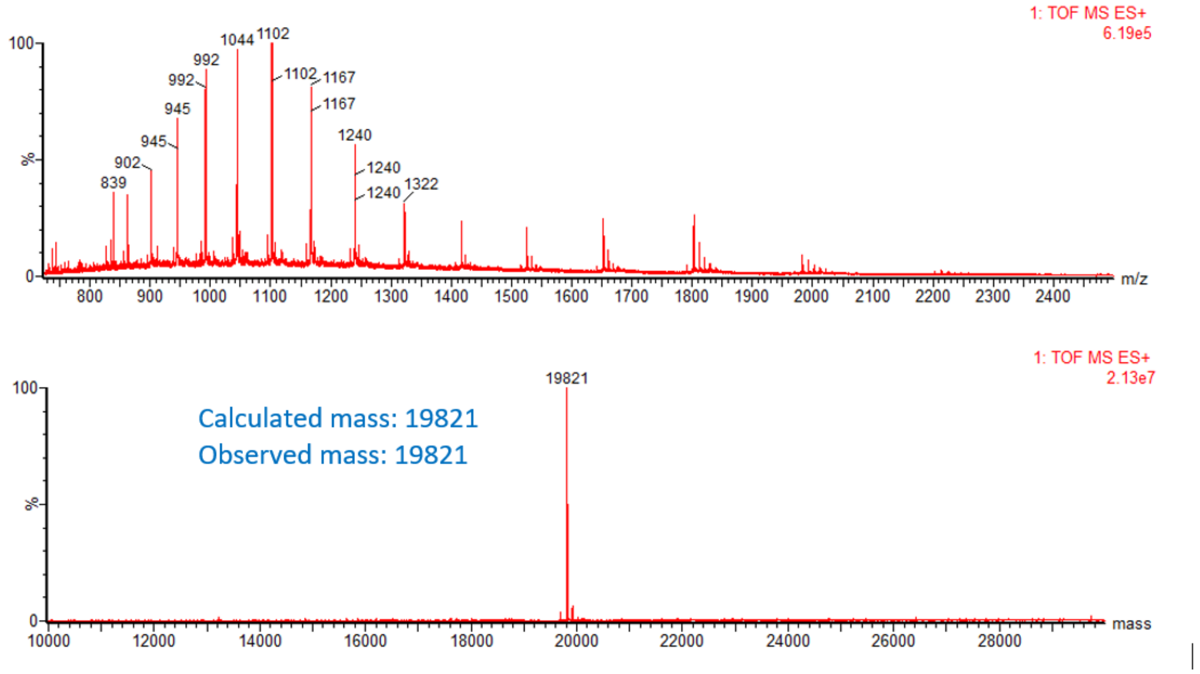

Figure S28. Total ion chromatogram and combined ion series and deconvoluted mass spectrum of the native antibody **DesAb-Aβ$_{3\text{-}9}$**.

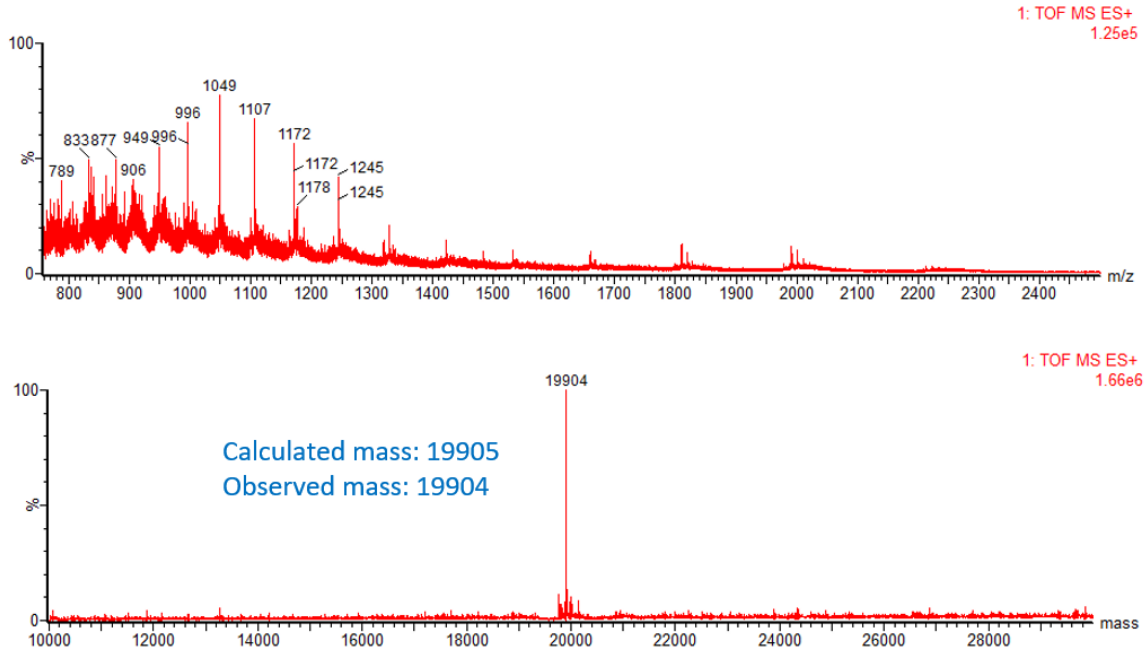

Figure S29. Total ion chromatogram and combined ion series and deconvoluted mass spectrum of the stapled antibody **DesAb-Aβ$_{3\text{-}9}$-1**.

10.3 Ellman's test of Oxetane stapled designed antibody

A 10 µL aliquot of oxetane stapled designed antibody (15 µL, 10 µM, 0.225 nmol) in 1X PBS buffer at pH 7.4 was transferred to a 0.5 mL eppendorf tube. Ellman's reagent (0.11 µL of a 20 mg/mL stock solution in H_2O, 5.63 nmol) was added and the resulting mixture vortexed for 30 seconds. After 6 h of additional shaking at room temperature, a 10 µL aliquot was analyzed by LC–MS and the stapled antibody (calculated mass, 19905; observed mass, 19905) was detected unaltered.

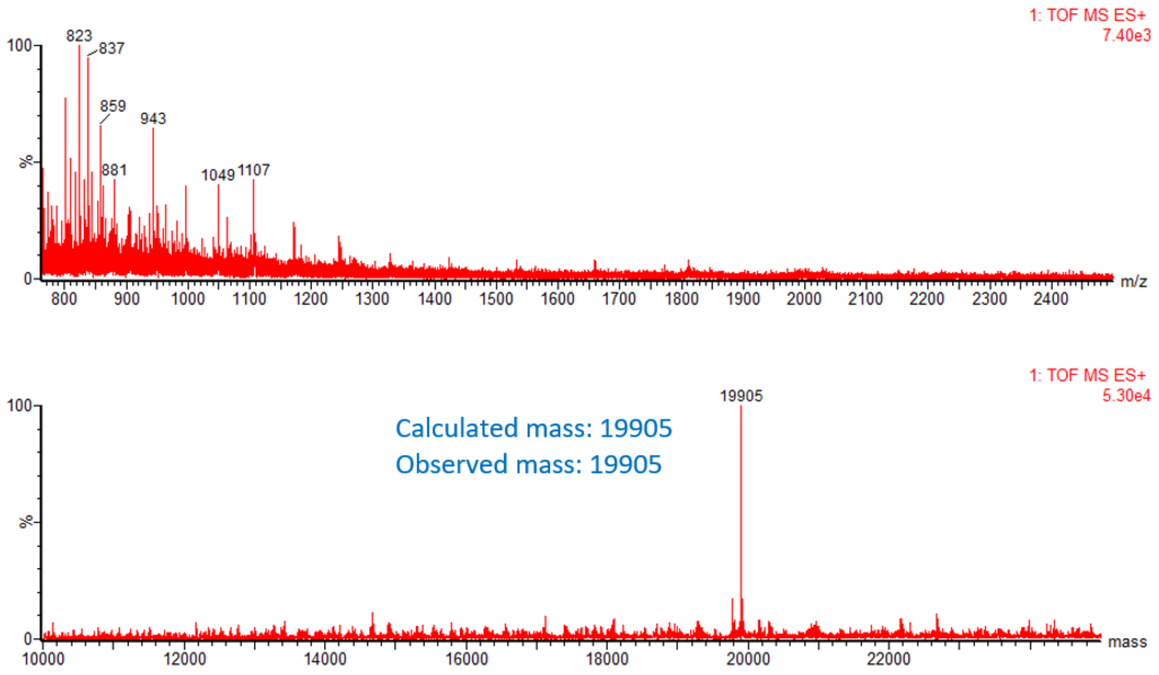

Figure S30. Total ion chromatogram and combined ion series and deconvoluted mass spectrum of the stapled antibody after Ellman's test.

10.4 Circular Dichroism spectroscopy of DesAb-Aβ$_{3-9}$ and DesAb-Aβ$_{3-9}$-1

The final concentration of the antibody samples was 10 µM in pure water. CD measurements were performed on an Aviv Model 410 spectrometer, which was routinely calibrated with (*1S*)-(+)-10-camphorsulfonic acid. Spectra were recorded at 298K with a 0.1 cm quartz cell over the wavelength range 250-200 nm at 50 nm·min^{-1}, with a bandwidth of 1.0 nm, the response time of 1 s, resolution step width of 1 nm and sensitivity of 20-50 Mdeg. Each spectrum represents the average of 3 scans.

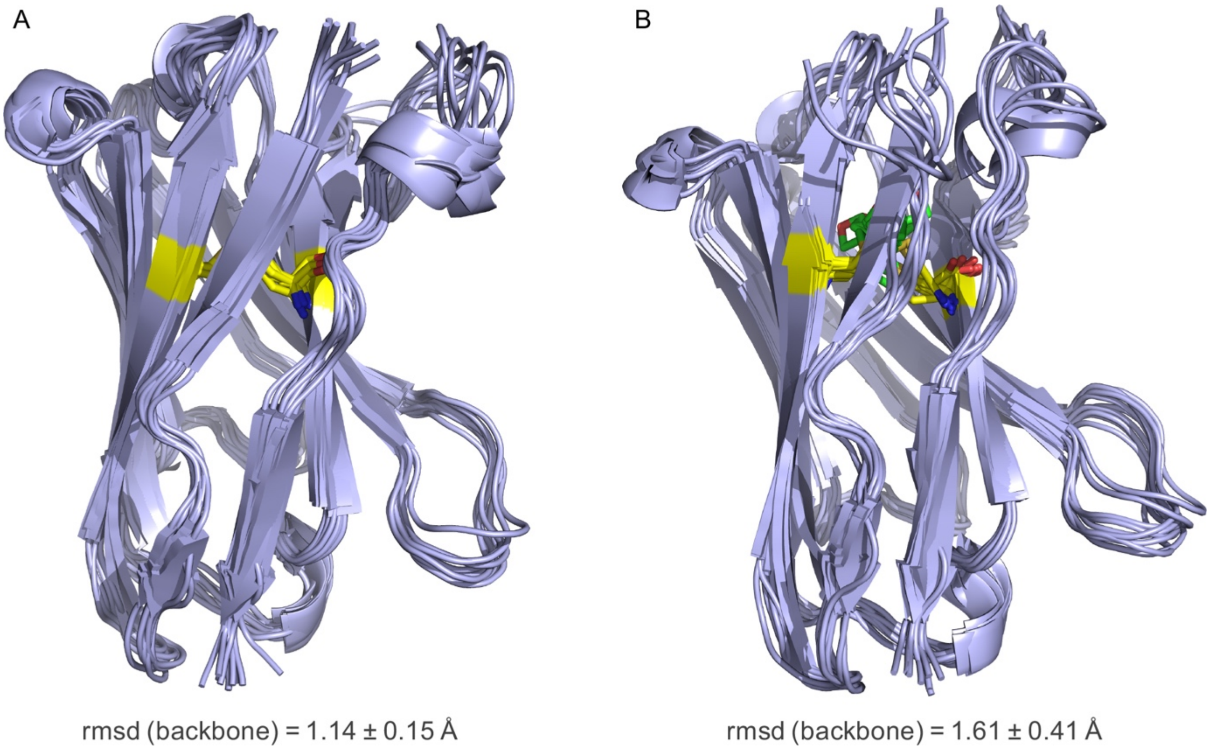

Figure S31. Structural ensembles obtained through 200 ns MD simulations for DesAb-Aβ$_{3-9}$ (A) and DesAb-Aβ$_{3-9}$-**1** (B). The numbers indicate the root-mean-square deviation (RMSD) for superimposition of the backbone with respect to the starting structure. 3D model of DesAb-Aβ3-9 was generated using the ABodyBuilder (http://opig.stats.ox.ac.uk/webapps/sabdab-sabpred/Modelling.php).

11. CRM_{197} stapling and activity studies

11.1 Preparation of the stapled CRM_{197}–1

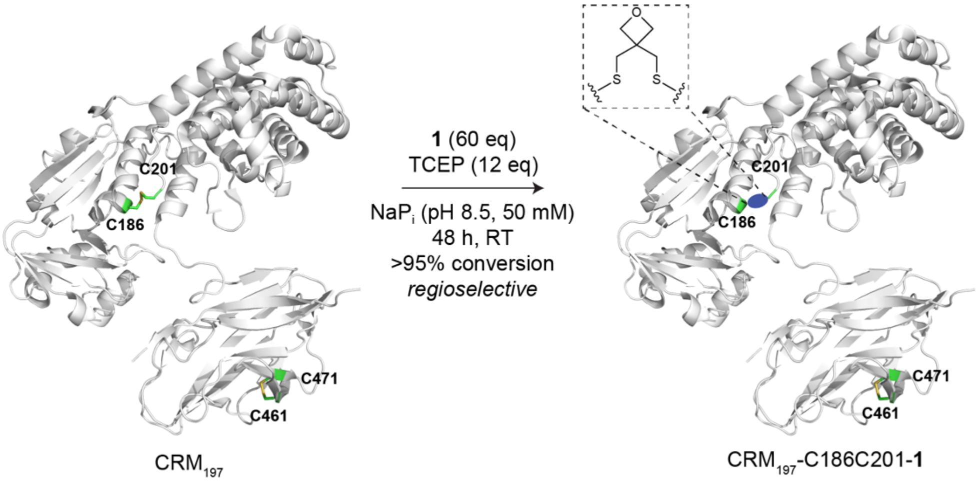

A solution of TCEP (0.05 M, 4.1 µL, 0.20 µmol) was added dropwise to a solution of CRM_{197} (5 mg/mL in 50 mM NaH_2PO_4 pH 8.5, 200 µL, 0.017 µmol). The resulting mixture was stirred at room temperature for 3.5 h under the dark, and then treated with 3,3-bis(bromomethyl)oxetane **1** (50 mg/mL in DMF, 5 µL, 1.02 µmol). The mixture was stirred for 48 h at room temperature, and then dialyzed against 50 mM NaH_2PO_4 pH 7.2 on Vivaspin with 10kDa cutoff membrane. LCMS calculated: 58498 Da; observed: [M+1] 58499 Da.

Sequence of CRM_{197}

GADDVVDSSKSFVMENFSSYHGTKPGYVDSIQKGIQKPKSGTQGNYDDDWKEFYST
DNKYDAAGYSVDNENPLSGKAGGVVKVTYPGLTKVLALKVDNAETIKKELGLSLTE
PLMEQVGTEEFIKRFGDGASRVVLSLPFAEGSSSVEYINNWEQAKALSVELEINFETR
GKRGQDAMYEYMAQACAGNRVRRSVGSSLSCINLDWDVIRDKTKTKIESLKEHGPI
KNKMSESPNKTVSEEKAKQYLEEFHQTALEHPELSELKTVTGTNPVFAGANYAAWA
VNVAQVIDSETADNLEKTTAALSILPGIGSVMGIADGAVHHNTEEIVAQSIALSSLMV
AQAIPLVGELVDIGFAAYNFVESIINLFQVVHNSYNRPAYSPGHKTQPFLHDGYAVS
WNTVEDSIIRTGFQGESGHDIKITAENTPLPIAGVLLPTIPGKLDVNKSKTHISVNGRKI
RMRCRAIDGDVTFCRPKSPVYVGNGVHANLHVAFHRSSSEKIHSNEISSDSIGVLGYQ
KTVDHTKVNSKLSLFFEIKS

Isotopically Averaged Molecular Weight = 58412.5039 Da

a.

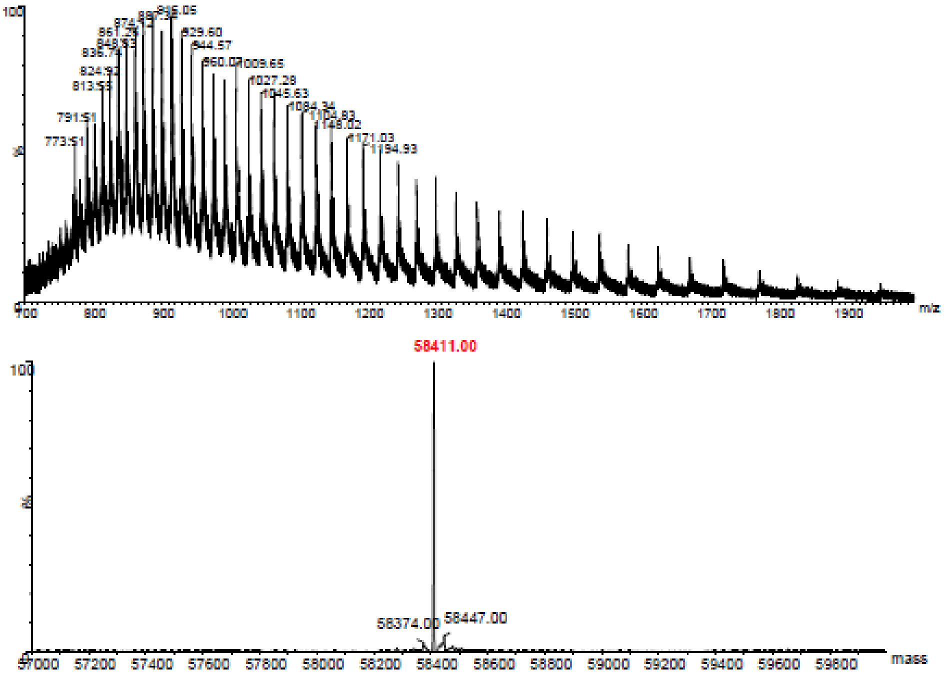

b.

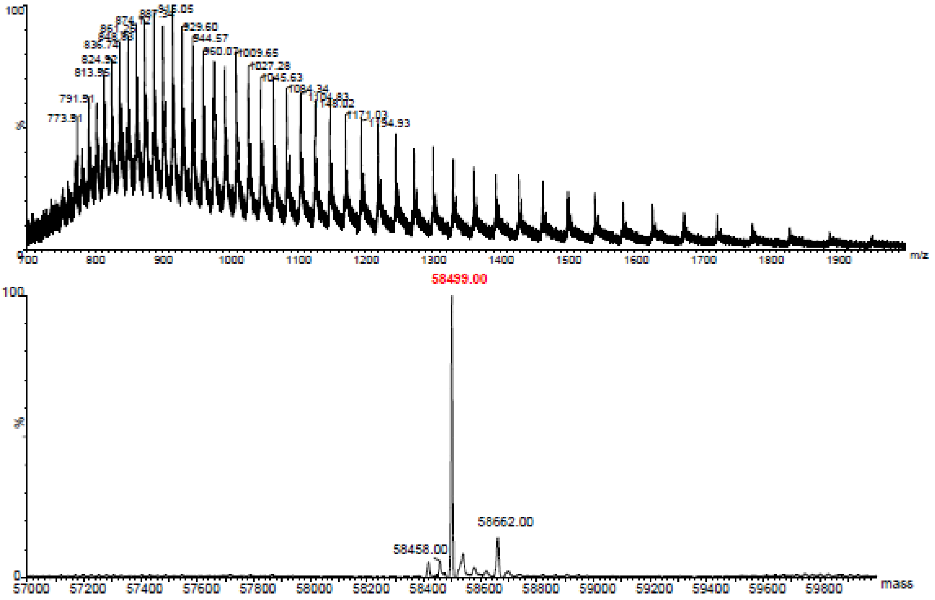

Figure S32. Combined ion series and deconvoluted mass spectrum of **a**, CRM_{197} and **b**, stapled CRM_{197}–**1**.

11.2 Tryptic digest and MS/MS analysis

Solutions were reduced (DTT) and alkylated (iodoacetamide) and subjected to enzymatic digestion with chymotrypsin overnight at 37 ºC. After digestion, the peptides were desalted, dried down, resuspended in 0.1% formic acid and pipetted into a sample vial and loaded onto an autosampler for automated LC-MS/MS analysis.

All LC-MS/MS experiments were performed using a Dionex Ultimate 3000 RSLC nanoUPLC (Thermo Fisher Scientific Inc, Waltham, MA, USA) system and a Q Exactive Orbitrap mass spectrometer (Thermo Fisher Scientific Inc, Waltham, MA, USA). Separation of peptides was performed by reverse-phase chromatography at a flow rate of 300 nL/min and a Thermo Scientific reverse-phase nano Easy-spray column (Thermo Scientific PepMap C18, 2µm particle size, 100A pore size, 75 µm i.d. x 50cm length). Peptides were loaded onto a pre-column (Thermo Scientific PepMap 100 C18, 5µm particle size, 100A pore size, 300 µm i.d. x 5mm length) from the Ultimate 3000 autosampler with 0.1% formic acid for 3 minutes at a flow rate of 10 µL/min. After this period, the column valve was switched to allow elution of peptides from the pre-column onto the analytical column. Solvent A was water + 0.1% formic acid and solvent B was 80% acetonitrile, 20% water + 0.1% formic acid. The linear gradient employed was 2-40% B in 30 minutes.

The LC eluant was sprayed into the mass spectrometer by means of an Easy-Spray source (Thermo Fisher Scientific Inc.). All *m/z* values of eluting ions were measured in an Orbitrap mass analyzer, set at a resolution of 70000 and was scanned between *m/z* 380-1500. Data dependent scans (Top 20) were employed to automatically isolate and generate fragment ions by higher energy collisional dissociation (HCD, NCE:25%) in the HCD collision cell and

measurement of the resulting fragment ions was performed in the Orbitrap analyser, set at a resolution of 17500. Singly charged ions and ions with unassigned charge states were excluded from being selected for MS/MS and a dynamic exclusion window of 20 seconds was employed.

Post-run, the data was processed using Protein Discoverer (version 2.1., ThermoFisher). Briefly, all MS/MS data were converted to mgf files and the files were then submitted to the Mascot search algorithm (Matrix Science, London UK) and searched against a custom database containing the CRM protein and common contaminant sequences (115 sequences, 38274 residues). Variable modifications of oxidation (M), deamidation (NQ) carbamidomethyl and the custom modification were applied. The peptide and fragment mass tolerances were set to 5ppm and 0.1 Da, respectively. A significance threshold value of $p<0.05$ and a peptide cut-off score of 20 were also applied.

The protein sequence of the target protein is:

GADDVVDSSKSFVMENFSSYHGTKPGYVDSIQKGIQKPKSGTQGNYDDDWKEFYST
DNKYDAAGYSVDNENPLSGKAGGVVKVTYPGLTKVLALKVDNAETIKKELGLSLTE
PLMEQVGTEEFIKRFGDGASRVVLSLPFAEGSSSVEYINNWEQAKALSVELEINFETR
GKRGQDAMYEYMAQACAGNRVRRSVGSSLSCINLDWDVIRDKTKTKIESLKEHGPI
KNKMSESPNKTVSEEKAKQYLEEFHQTALEHPELSELKTVTGTNPVFAGANYAAWA
VNVAQVIDSETADNLEKTTAALSILPGIGSVMGIADGAVHHNTEEIVAQSIALSSLMV
AQAIPLVGELVDIGFAAYNFVESIINLFQVVHNSYNRPAYSPGHKTQPFLHDGYAVS
WNTVEDSIIRTGFQGESGHDIKITAENTPLPIAGVLLPTIPGKLDVNKSKTHISVNGRKI
RMRCRAIDGDVTFCRPKSPVYVGNGVHANLHVAFHRSSSEKIHSNEISSDSIGVLGYQ
KTVDHTKVNSKLSLFFEIKS

Chymotrypsin was used as the enzyme to try and generate a peptide which incorporated both cysteines which were thought to bind the modification (highlighted). If this peptide was generated during the digestion, it would have been ideal size for sequencing by MS/MS analysis. Chymotrypsin can also cleave at leucine residues (as well as Y, F and W) and it

appears from the Mascot data, that the peptide of interest has been cleaved at the second leucine residue (shown underlined below).

Protein sequence coverage: 76%

Matched peptides shown in ***bold red***.

```
  1 GADDVVDSSK SFVMENFSSY HGTKPGYVDS IQKGIQKPKS GTQGNYDDDW
 51 KEFYSTDNKY DAAGYSVDNE NPLSGKAGGV VKVTYPGLTK VLALKVDNAE
101 TIKKELGLSL TEPLMEQVGT EEFIKRFGDG ASRVVLSLPF AEGSSSVEYI
151 NNWEQAKALS VELEINFETR GKRGQDAMYE YMAQACAGNR VRRSVGSSLS
201 CINLDWDVIR DKTKTKIESL KEHGPIKNKM SESPNKTVSE EKAKQYLEEF
251 HQTALEHPEL SELKTVTGTN PVFAGANYAA WAVNVAQVID SETADNLEKT
301 TAALSILPGI GSVMGIADGA VHHNTEEIVA QSIALSSLMV AQAIPLVGEL
351 VDIGFAAYNF VESIINLFQV VHNSYNRPAY SPGHKTQPFL HDGYAVSWNT
401 VEDSIIRTGF QGESGHDIKI TAENTPLPIA GVLLPTIPGK LDVNKSKTHI
451 SVNGRKIRMR CRAIDGDVTF CRPKSPVYVG NGVHANLHVA FHRSSSEKIH
501 SNEISSDSIG VLGYQKTVDH TKVNSKLSLF FEIKS
```

Discussion

This means that the linked modification between the two cysteines would be bridging two different peptides and in effect, we would have two peptides which were cross-linked by the modification. This would make it very difficult to get any useful data from Mascot, because we are searching single peptide sequences, rather than two peptide sequences. The resulting MS/MS data would probably be a combination of two overlapping sets of fragment ions and this could lead to poor matches when searched by Mascot. Also, because the modification is a bound to two different residues, even if we did generate a single peptide, the MS/MS data could still prove to be very complex. It may be, that little sequence information is generated because the peptide fragments in an unusual way as a result of cleavage at the modification, or cleavage at the two cysteine residues. No modifications were detected at lysine residues, but again, the MS/MS data could be very complex given the number of lysine residues in the protein sequence.

11.3 Characterization in vitro and in vivo of CRM_{197}–1

Differential scanning calorimetry (DSC)

Samples were diluted in 10 mM sodium phosphate buffer pH 7.2 to a final protein concentration of 0.2 mg/mL. DSC data were collected with a Micro-Cal VP-Capillary DSC instrument (GE Healthcare) with integrated autosampler. Samples (500 µL) were transferred to a 96-well plate and kept in the instrument autosampler at 5 ºC until analysis. A single DSC scan was recorded for each sample in the temperature range of 10–110 ºC with a thermal ramping of 150 ºC/h and a 5s filter period. Data were analyzed after subtraction of the reference data from a sample containing only buffer, using the Origin 7 software (OriginLab).

Circular dichroism (CD)

Far-UV: CRM_{197} and CRM_{197}-**1** proteins were prepared in 10 mM sodium phosphate buffer pH 7.2 at a final protein concentration of 0.2 mg/mL. Far-UV CD spectra were obtained with a J-810 spectropolarimeter (Jasco) at 20 ± 1 ºC. Far-UV CD spectra were recorded in the range 180–260 nm in a 1 mm optical path length quartz cuvette, at 1 nm bandwidth, 0.2 nm step size, 4 s response time, with a speed of 10 nm/min and 6 scans. All spectra were subtracted of the baseline (buffer) and converted into molar ellipticity ([θ], $deg \cdot cm^2 \cdot dmol^{-1} \cdot residue^{-1}$), by using a mean residue molecular mass of 109 Da.

Near-UV: CRM_{197} and CRM_{197}-**1** proteins were prepared in 5 mM potassium phosphate buffer pH 7.4 at a final protein concentration of 1.0 mg/mL. Near-UV CD spectra were obtained with a J-810 spectropolarimeter (Jasco) at 20 ± 1 ºC. Near-UV CD spectra were recorded in the range 320–250 nm in a 1 mm optical path length quartz cuvette, at 1 nm bandwidth, 0.5 nm step size, 2 s response time, with a speed of 10 nm/min and 6 scans. All spectra were subtracted

of the baseline (buffer) and converted into molar ellipticity ([θ], deg·cm^2·dmol^{-1}·residue^{-1}), by using a mean residue molecular mass of 109 Da.

Immunogenicity in Mice

Eight female BALB/c mice were immunized by subcutaneous injection of 5μg in protein content of CRM_{197}. Mice received the vaccines at days 1, 14 and 28. Sera were bled at days 1, 27 and 42.

ELISA analysis

Microtiter plates (96 wells, NUNC, Maxisorp) were coated with 100 μL of CRM_{197} in PBS pH 7.4 (2 μg/mL). Plates were incubated overnight at 2-8 °C, washed three times with PBS pH 7.4 and 0.05% Tween-20 (Sigma), and saturated by incubating with 250 μL/well of PBS-2% BSA-0.05% Tween-20 for 1 hour at 37°C. Two-fold serial dilutions of test and standard sera in PBS-2% BSA-0.05% Tween-20 were added to each well. Plates were then incubated at 37°C for 2 hours, washed with PBS pH 7.4 and 0.05% Tween-20, and incubated for 1 additional hour at 37°C with either anti-mouse IgG-alkaline phosphatase (Sigma) diluted 1:2000 in PBS-2% BSA-0.05% Tween. After washing, the plates were developed with a solution of p-nitrophenylphosphate (p-NPP, 4mg/mL) (Sigma) diluted in 1M diethanolamine (DEA) (Sigma) pH 9.8, at room temperature for 30 minutes. Enzymatic activity was stopped with 7% EDTA, and the absorbance is measured using a plate reader (SPECTRAmax) with wavelength set at 405 nm. ELISA titer was espressed as the reciprocal serum dilution leading to OD 1.0.

For competitive ELISA, Serial tenfold dilutions of the inhibitors were made in TPBS separately, and 50 μL of pre-diluted mAb (Anti-Diphtheria Toxin antibody IGX3492 from

ABCAM) or pAb, were added to the coated plate and immediately mixed with 50 µL of inhibitor pre-diluted solution. Then the procedure described above was followed.

For avidity ELISA the procedure described above was followed, with the inclusion of an extra step.[13] After the plate was washed following incubation of the dilution of pooled polyclonal serum from each immunized group, ammonium thiocyanate (NH_4SCN) phosphate buffer, pH 6.0, was added to the appropriate wells in triplicate, in a final concentration of 8 M and dilutions thereof. The plates were allowed to stand for 15 min at room temperature before triple washing with TPBS and proceeding with the assay as described above. For the data analysis, the $\log_{10}$ of binding % was plotted vs the molar concentrations of NH_4SCN. The data, which all presented an OD >1, were fitted by linear regression. Sets of data having a linear fitting with a correlation coefficient ≥ 0.88 M were accepted. The NH_4SCN concentration able to produce a 50% reduction in the initial IgG level was calculated (avidity index, AI). Standard deviation for the triplicate sets of data was calculated.

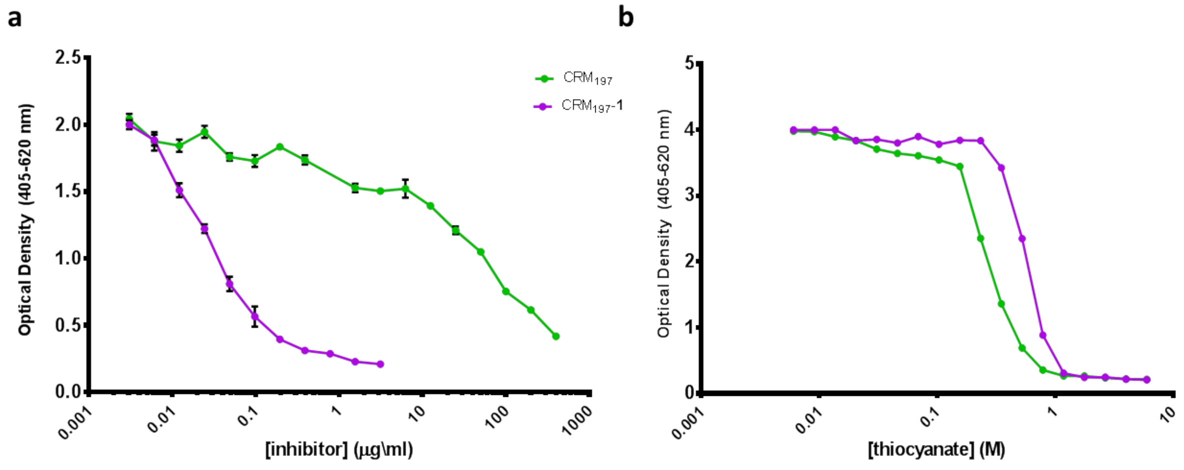

Figure S33. a. Inhibition of commercial anti DT (mutated G52E, corresponding to CRM_{197}) recombinant human mAb; **b.** Thiocyanate elution curves at different salt dilutions.

12. ^{1}H and ^{13}C NMR spectra

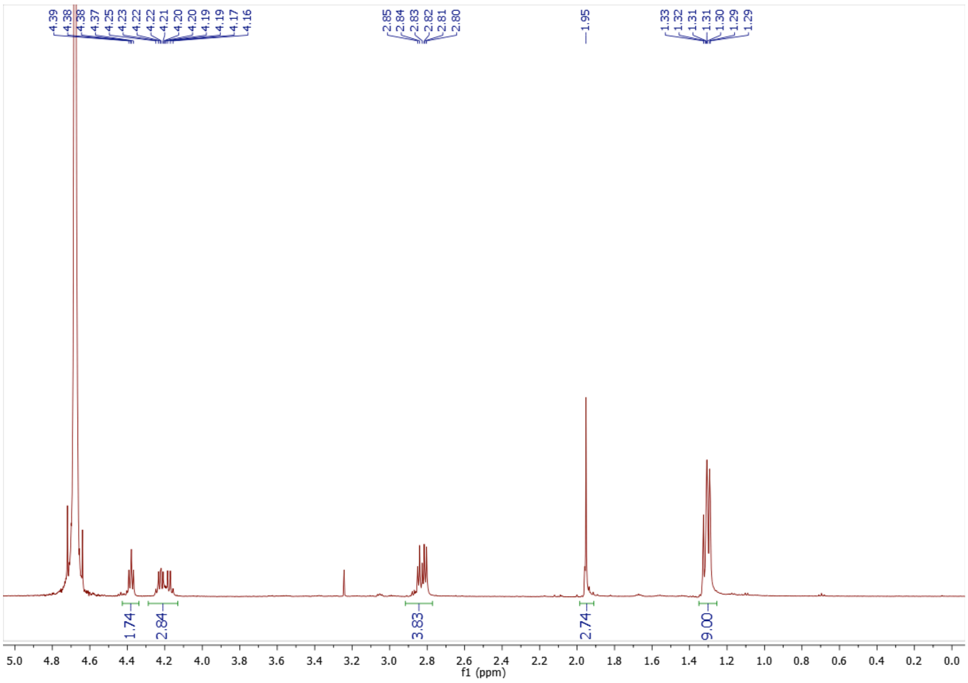

Figure S34. ^{1}H NMR (500 MHz, D_2O) of peptide **CAAAC**.

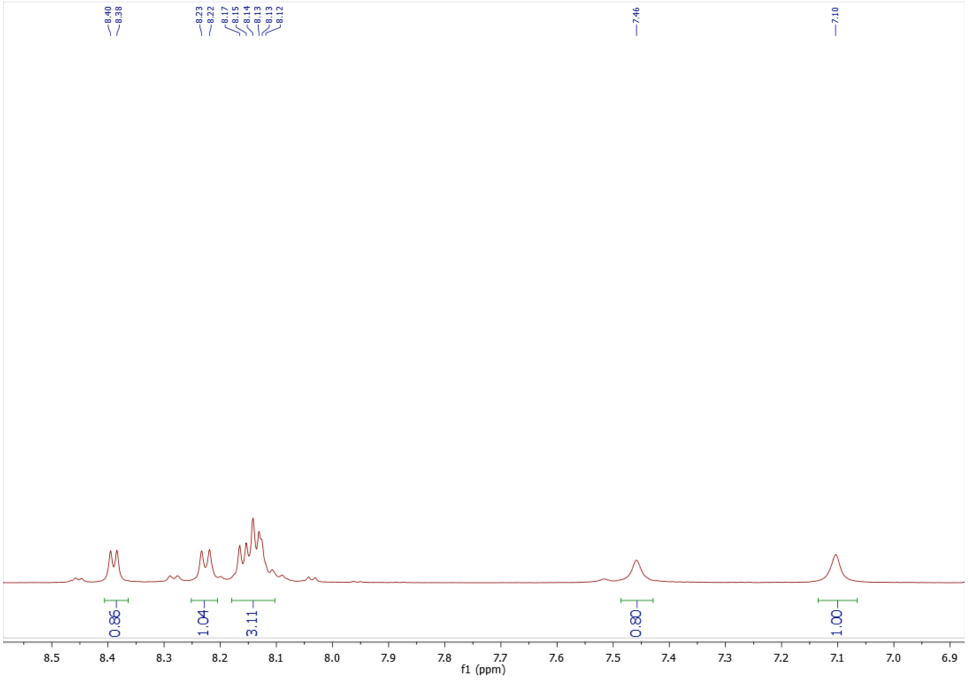

Figure S35. ^{1}H NMR (500 MHz, H_2O/D_2O (9:1)) of peptide **CAAAC**.

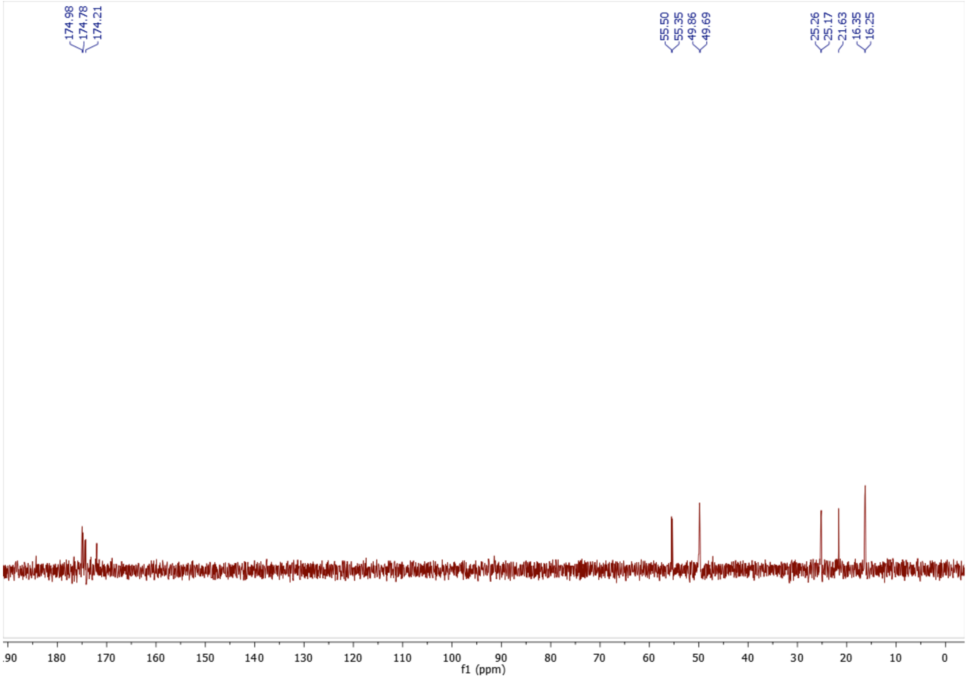

Figure S36. ^{13}C NMR (126 MHz, D_2O) of peptide **CAAAC**.

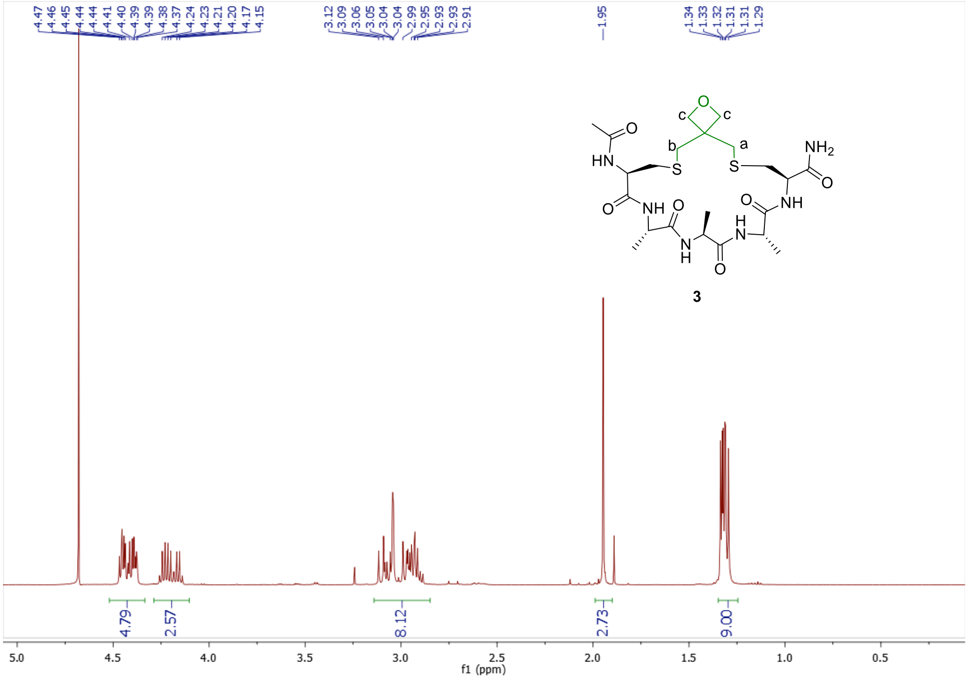

Figure S37. ^{1}H NMR (500 MHz, D_2O) of stapled peptide **CAAAC-1**.

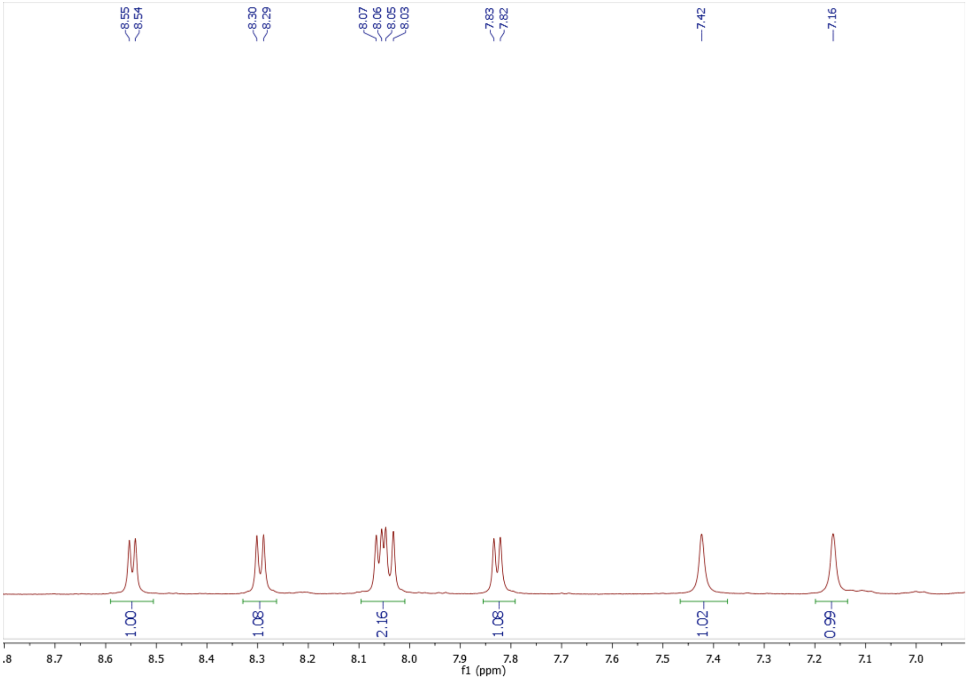

Figure S38. ^{1}H NMR (500 MHz, H_2O/D_2O (9:1)) of stapled peptide **CAAAC-1**.

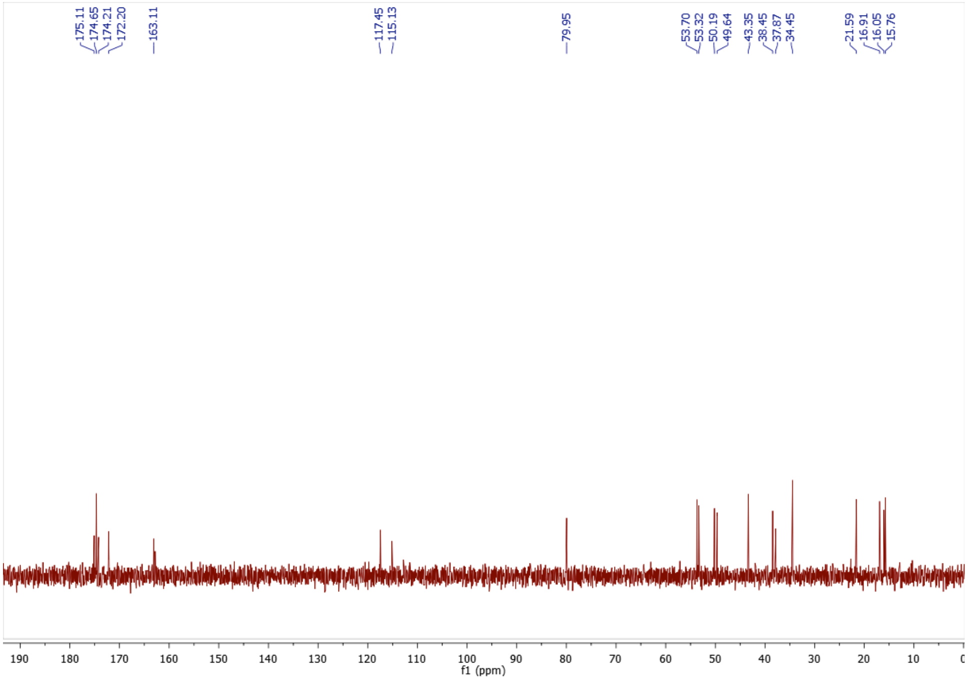

Figure S39. ^{13}C NMR (126 MHz, D_2O) of stapled peptide **CAAAC-1**.

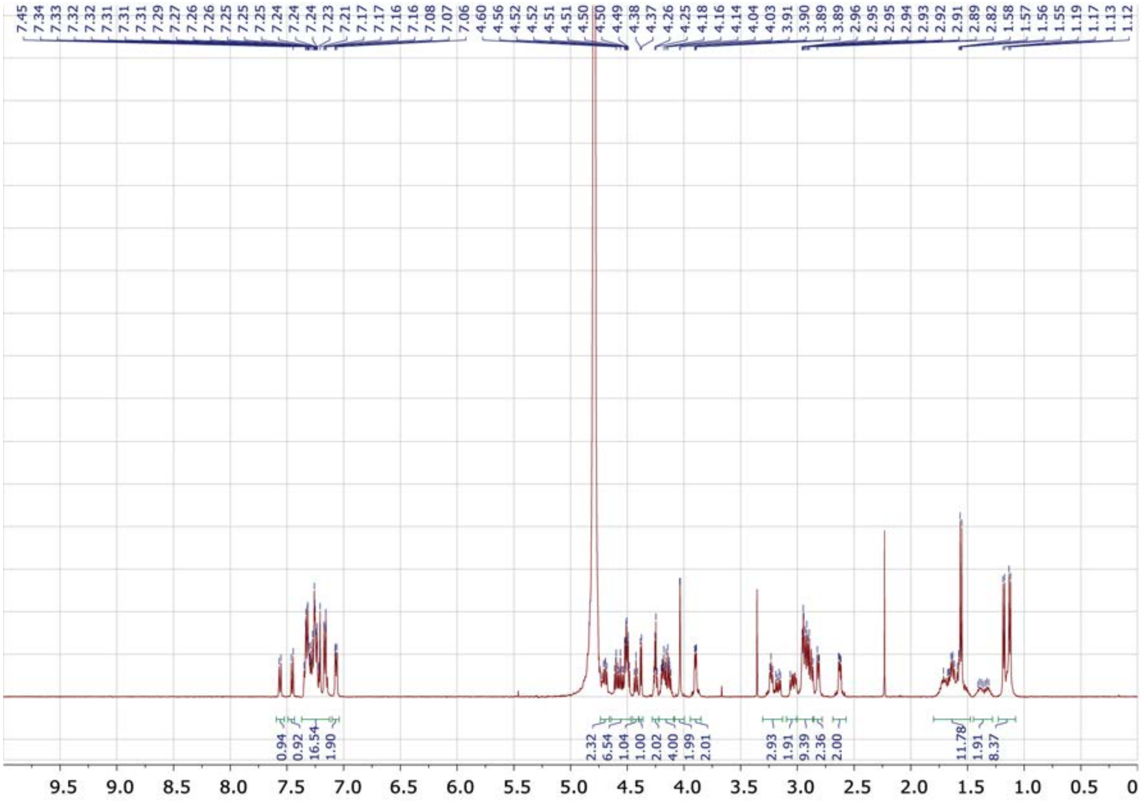

Figure S40. ^{1}H NMR 500 MHz in D_2O registered at 298K of somatostatin **2**.

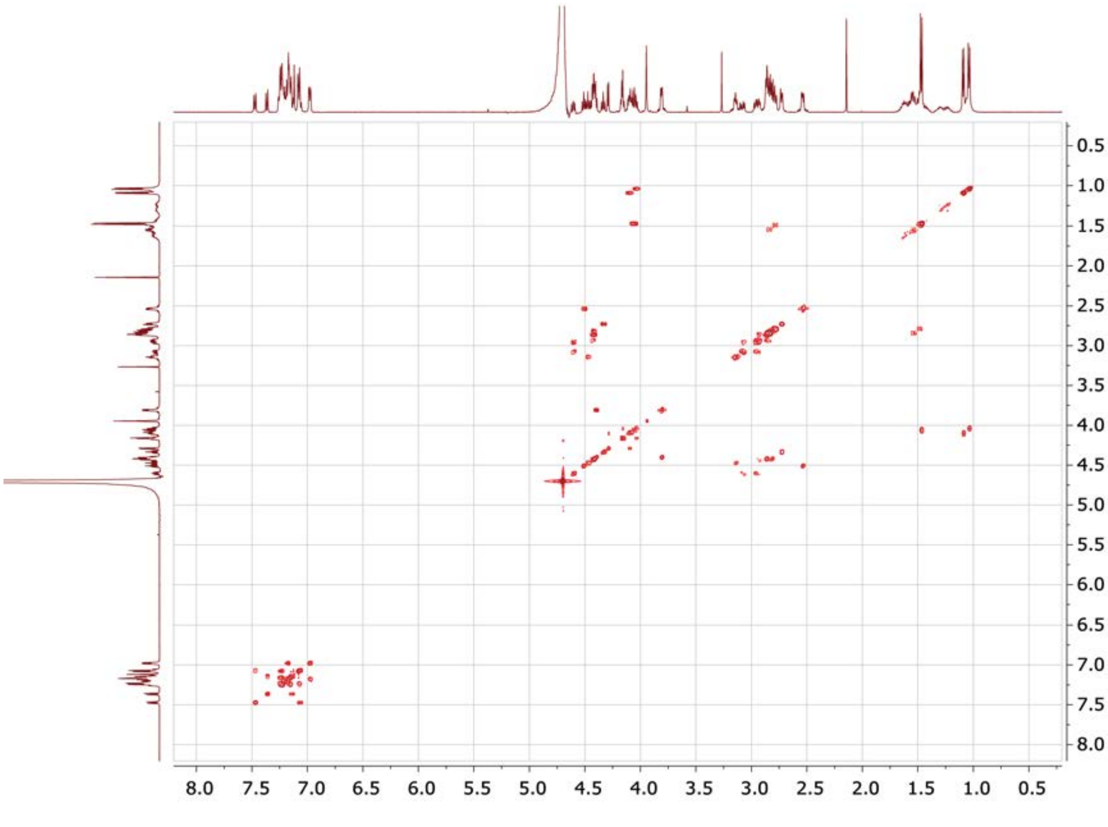

Figure S41. COSY in D_2O registered at 298K of somatostatin **2**.

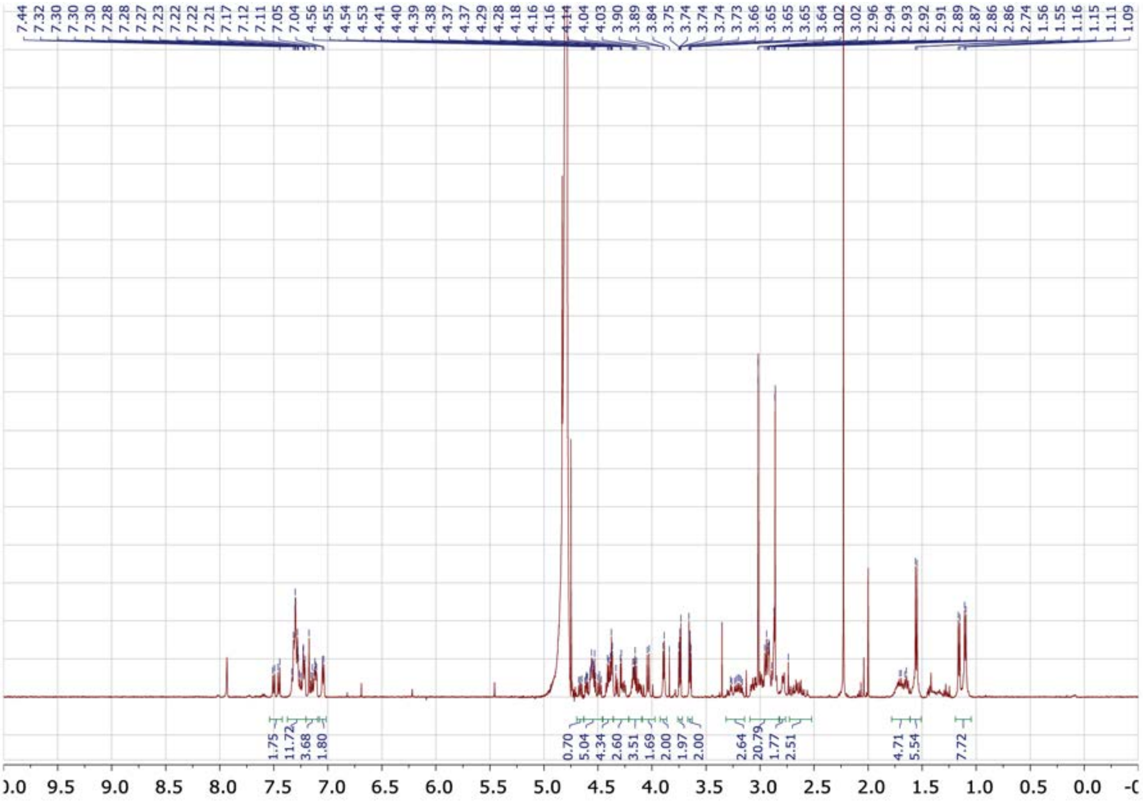

Figure S42. ^{1}H NMR 500 MHz in D_2O registered at 298K of stapled somatostatin **3**.

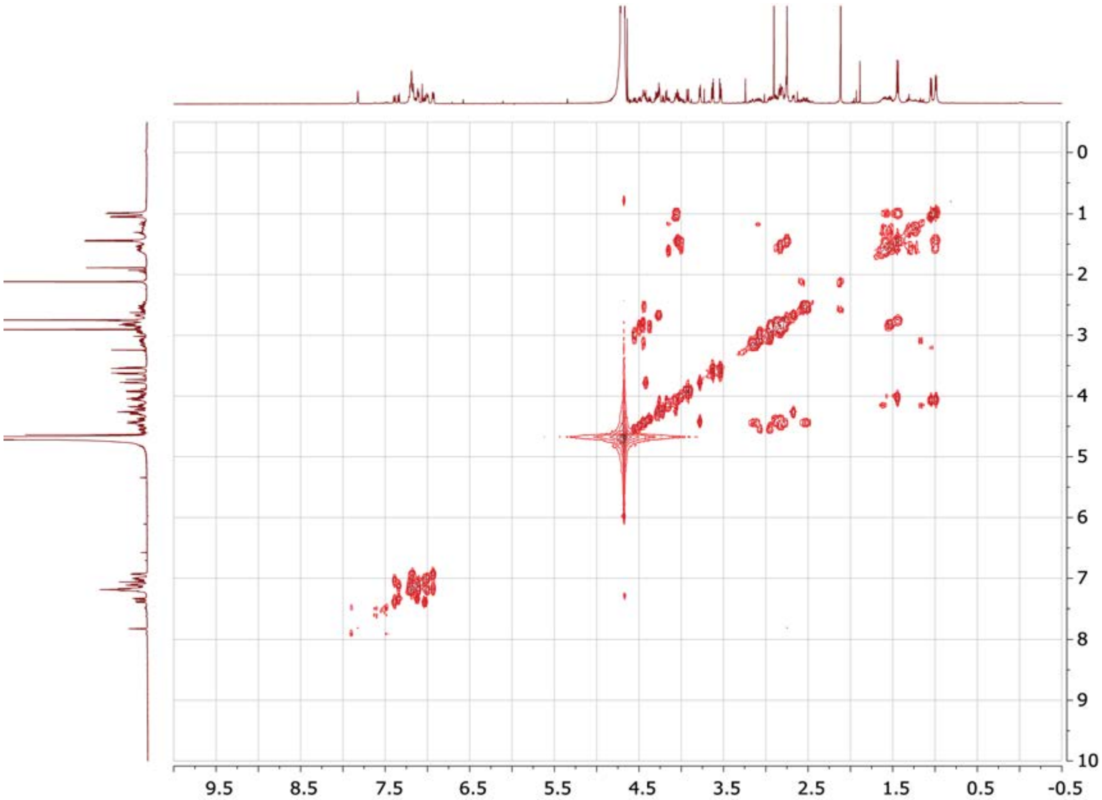

Figure S43. COSY in D_2O registered at 298K of stapled somatostatin **3**.

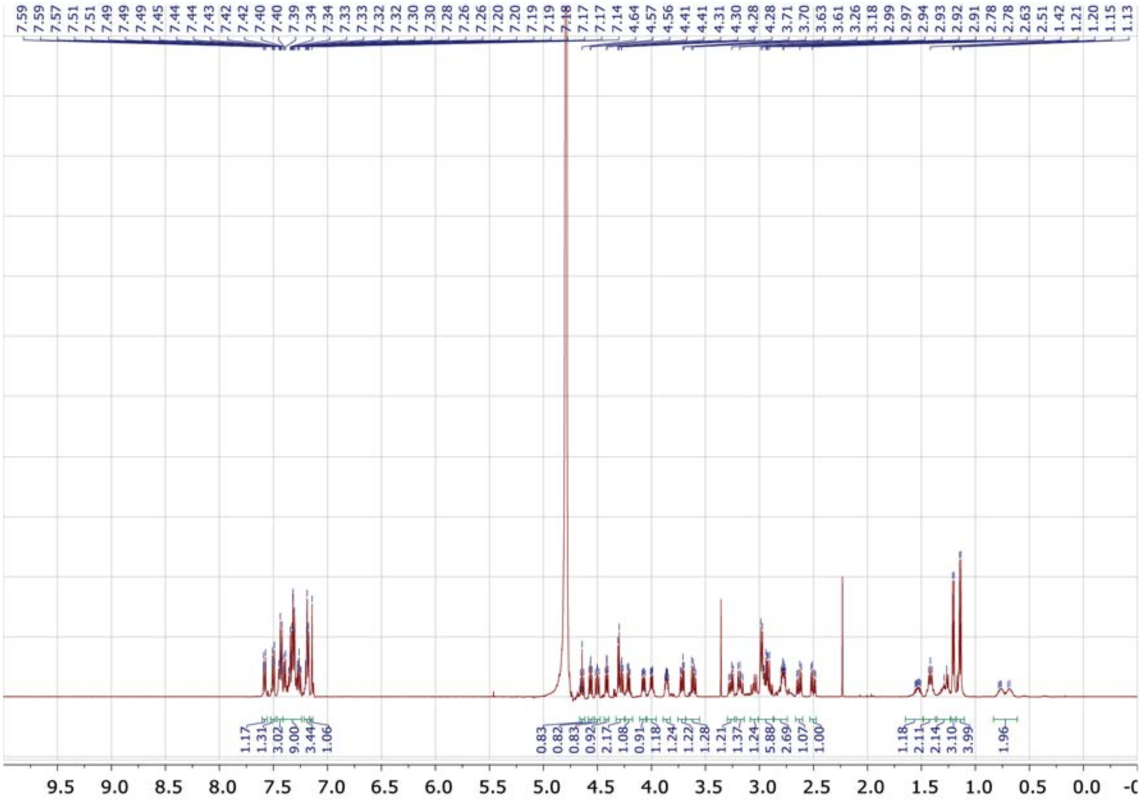

Figure S44. ^{1}H NMR 500 MHz in D_2O registered at 298K of octreotide **4**.

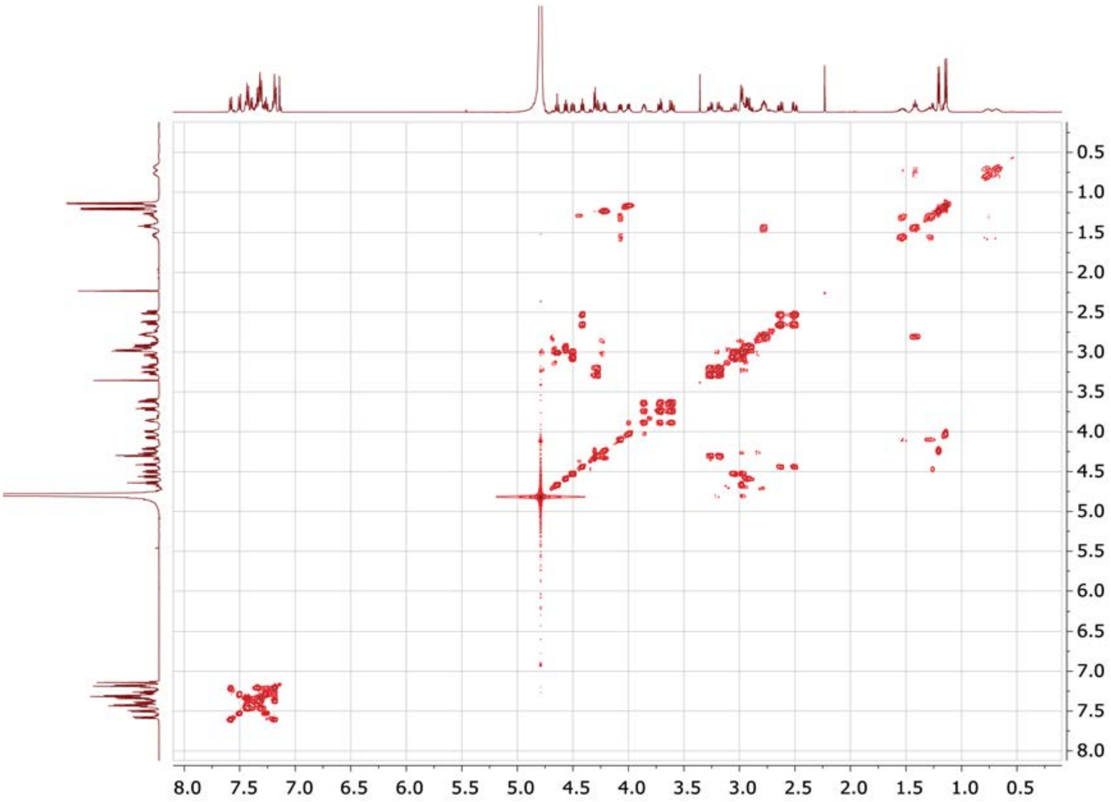

Figure S45. COSY in D_2O registered at 298K of octreotide **4**.

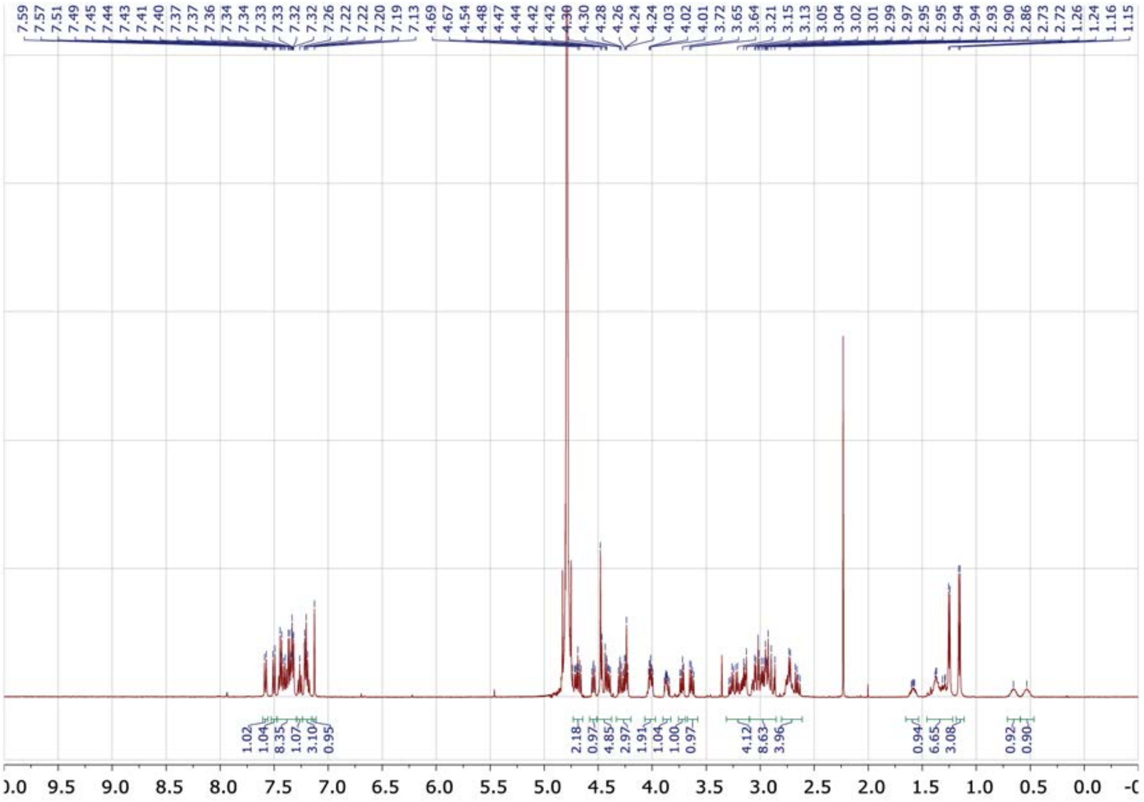

Figure S46. ^{1}H NMR 500 MHz in D_2O registered at 298K of stapled octreotide **5**.

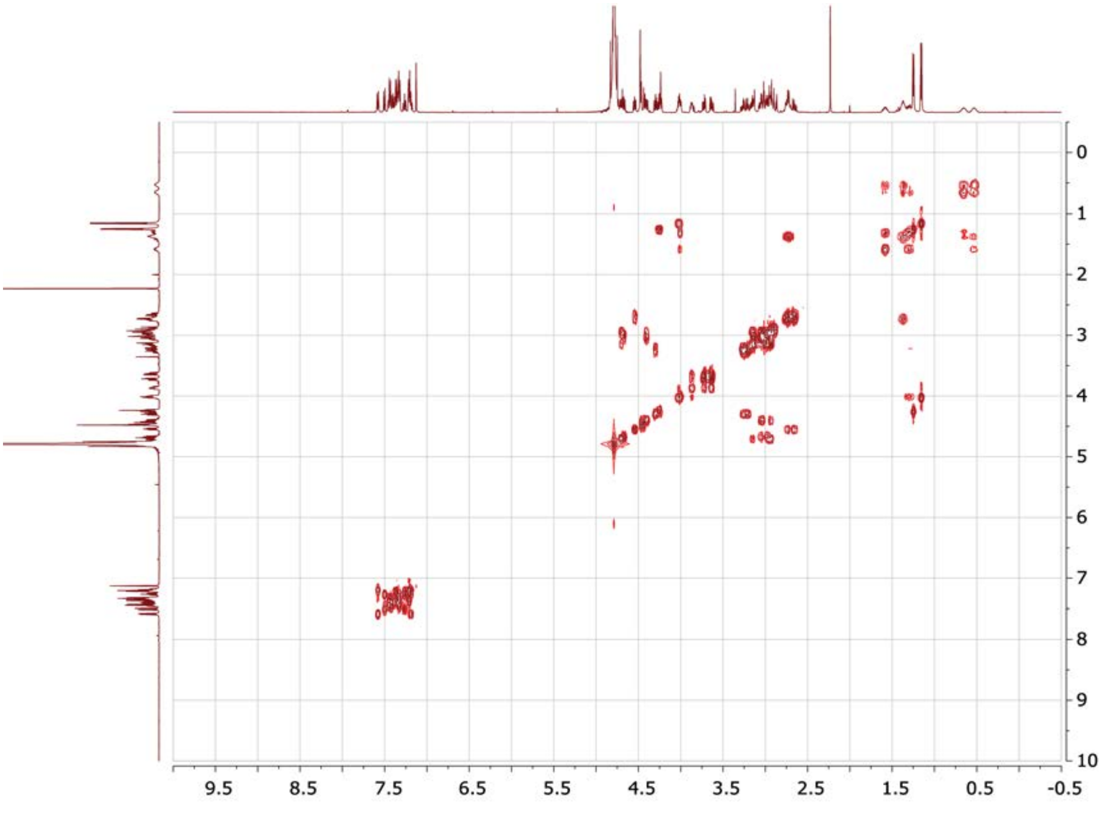

Figure S47. COSY in D_2O registered at 298K of stapled octreotide **5**.

13. HPLC chromatograms

See HPLC details in 'Synthesis section'.

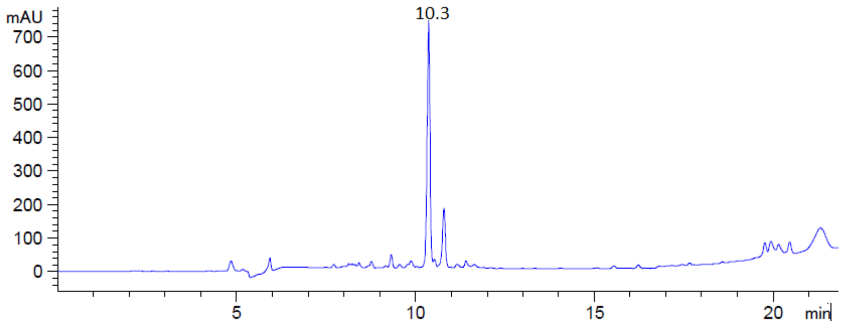

Figure S48. Purification of peptide **CAAAC** by HPLC.

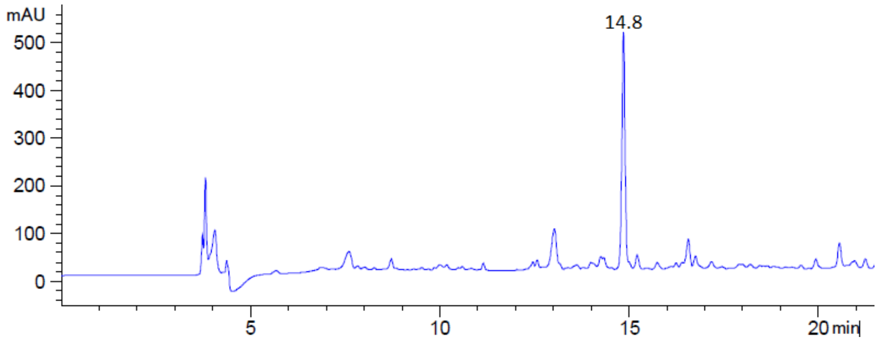

Figure S49. Purification of peptide **CAAAC-1** by HPLC.

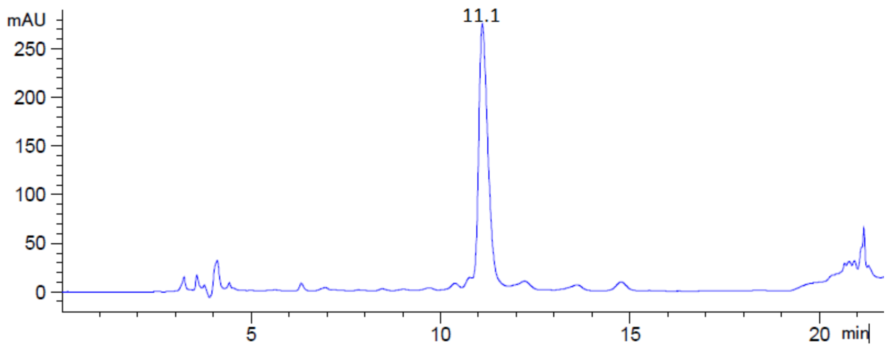

Figure S50. Purification of peptide **4** by HPLC.

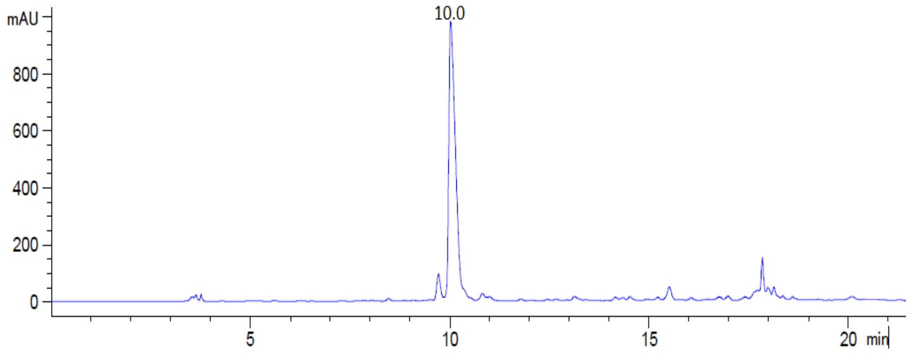

Figure S51. Purification of peptide **5** by HPLC.

14. References

[1] R. Subirós-Funosas, R. Prohens, R. Barbas, A. El-Faham, F. Albericio, *Chem. Eur. J.* **2009**, *15*, 9394–9403.

[2] D. A. Case, e. al., *AMBER 2016, University of California, San Francisco* **2012**.

[3] V. Hornak, R. Abel, A. Okur, B. Strockbine, A. Roitberg, C. Simmerling, *Proteins* **2006**, *65*, 712–725.

[4] K. T. Debiec, D. S. Cerutti, L. R. Baker, A. M. Gronenborn, D. A. Case, L. T. Chong, *J. Chem. Theory Comput.* **2016**, *12*, 3926–3947.

[5] C. I. Bayly, P. Cieplak, W. Cornell, P. A. Kollman, *J. Phys. Chem.* **1993**, *97*, 10269–10280.

[6] M. J. Frisch, e. al., *Gaussian, Inc.* **2009**.

[7] T. A. Andrea, W. C. Swope, H. C. Andersen, *J. Chem. Phys.* **1983**, *79*, 4576–4458.

[8] T. Darden, D. York, L. Pedersen, *J. Chem. Phys.* **1993**, *98*, 10089–10092.

[9] a) G. Lautrette, F. Touti, H. G. Lee, P. Dai, B. L. Pentelute, *J. Am. Chem. Soc.* **2016**, *138*, 8340–8343; b) S. P. Brown, A. B. Smith, *J. Am. Chem. Soc.* **2015**, *137*, 4034–4037; c) S. Kalhor-Monfared, M. R. Jafari, J. T. Patterson, P. I. Kitov, J. J. Dwyer, J. M. Nuss, R. Derda, *Chem. Sci.* **2016**, *7*, 3785–3790.

[10] F. Corzana, J. H. Busto, S. B. Engelsen, J. Jiménez-Barbero, J. L. Asensio, J. M. Peregrina, A. Avenoza, *Chem. Eur. J.* **2006**, *12*, 7864–7871

[11] G. Melacini, Q. Zhu, M. Goodman, *Biochemistry* **1997**, *36*, 1233–1241.

[12] X. Yang, K. Ma, *J. Bacteriol.* **2010**, *192*, 1370–1376.

[13] R. A. Macdonald, C. S. Hosking, C. L. Jones, *J. Immunol. Methods* **1988**, *10*, 191–194.
